# Material‐Gradient Enabled Enhancement of Strength and Strain‐Hardening of Lattice Structures

**DOI:** 10.1002/advs.202511185

**Published:** 2025-10-07

**Authors:** Junhao Ding, Yaojie Wen, Qianhua Wang, Shuo Qu, Xu Song, Baicheng Zhang

**Affiliations:** ^1^ Beijing Advanced Innovation Center for Materials Genome Engineering Institute for Advanced Materials and Technology University of Science and Technology Beijing Beijing 100083 P. R. China; ^2^ Department of Mechanical and Automation Engineering Chinese University of Hong Kong Shatin Hong Kong 999077 P. R. China; ^3^ Beijing laboratory of modern transportation metal materials and processing technology University of Science and Technology Beijing Beijing 100083 P. R. China

**Keywords:** freely controlled material distribution, material‐gradient engineering, multi‐material printing, tailored mechanical properties

## Abstract

Additive manufacturing enables the creation of architected lattices with tailored mechanical responses, yet conventional geometry‐driven designs often suffer from structural redundancy and limited adaptability. Here, spatially continuous material gradients are introduced into body‐centered cubic (BCC) truss and Gyroid triply periodic minimal surface (TPMS) lattices using a custom laser powder bed fusion platform capable of alloy composition control. This strategy decouples mechanical tuning from geometry, enabling programmable deformation through material distribution alone. Compression experiments reveal that continuous gradients fundamentally reconfigure the collapse mechanism, guiding a stable plastic front from softer to stiffer regions. This controlled strain propagation enhances structural performance, increasing plateau stress and energy absorption by up to 23.6% and 25.4% in BCC lattices, and 9.8% and 12% in Gyroid structures, respectively, compared to sharp‐interface counterparts. These improvements arise from suppressed localization and stabilized plastic flow, effectively imparting an emergent strain‐hardening behavior at the macroscale, even in base alloys with limited intrinsic hardening. These findings establish material gradients as a powerful design axis for metamaterials, advancing beyond passive shape optimization toward active, composition‐driven performance control.

## Graphical abstract

This study employs material gradients in lattice structures via alloy‐controlled additive manufacturing, decoupling mechanical behavior from geometry. Gradients guide stable plastic fronts, enhancing plateau stress and energy absorption by up to 25.4%. The approach suppresses failure localization and induces strain hardening, establishing composition‐driven control as a new mechanism for strengthening architected structures.

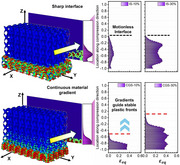

## 1 Introduction

Nature provides remarkable examples of optimized design through the strategic selection of compositional and functional gradients in both materials and structures.^[^ ^1^, ^2^, ^3^, ^4^ ^]^ Lattice structures, defined by their periodic cellular architecture, have emerged as a transformative class of metamaterials in modern engineering.^[^ ^5^, ^6^ ^]^ These architected materials exhibit an exceptional combination of properties, including high strength,^[^ ^7^ ^]^ stiffness,^[^ ^8^ ^]^ and energy absorption,^[^ ^9^, ^10^ ^]^ while maintaining a lightweight profile. Recent advancements in additive manufacturing (AM) have unlocked larger design freedom, enabling the fabrication of diverse and complex structures tailored for specific applications. Achieving material‐structure‐performance integrated AM represents a paradigm shift toward the production of high‐performance components.

The current approach to adjusting the mechanical properties of lattice structures largely revolves around geometric design by manipulating parameters such as unit cell size, topology, and diameter/thickness.^[^ ^11^, ^12^, ^13^ ^]^ For example, functionally graded sheet‐based lattices, fabricated by AM, have demonstrated improved shear failure resistance by introducing wall thickness gradients perpendicular to the loading direction.^[^ ^13^ ^]^ Similarly, topological gradients in triply periodic minimal surface (TPMS) structures have enabled predictable layer‐by‐layer deformation, leading to enhanced strength and energy absorption.^[^ ^14^ ^]^ Heterogeneous lamellar lattices, designed by spatially varying lattice types, have inhibited shear band nucleation, resulting in significant strengthening effects.^[^ ^15^ ^]^ However, these geometry‐based methods often involve trade‐offs between design complexity and manufacturability,^[^ ^16^ ^]^ such as compromised dimensional accuracy or increased weight. Furthermore, the inherent limitations of AM, including limited printing fidelity and an adjustable range of geometric factors, make it challenging to adjust the properties across a wide range. Importantly, relying solely on geometric manipulation limits the ability to synergistically optimize other material properties, such as electrical conductivity, thermal stability, and chemical resistance, leading to unnecessary design trade‐offs.

One promising avenue is the multi‐material design strategy,^[^ ^17^, ^18^, ^19^, ^20^ ^]^ which introduces material gradients to tailor properties without altering geometry.^[^ ^21^ ^]^ It has been demonstrated that the material gradients can alleviate stress concentrations, delaying crack propagation,^[^ ^22^ ^]^ enhancing toughness,^[^ ^23^ ^]^ and expanding mechanical tunability with great tailoring space beyond monolithic material limits. For instance, coatings and infiltration techniques have been used to combine soft, tough materials with hard, brittle ones, achieving exceptional combinations of strength and energy absorption.^[^ ^23^, ^24^ ^]^ However, these approaches often lack precise control over material distribution. Recent advancements in multi‐material 3D printing have enabled control over material gradients,^[^ ^25^ ^]^ particularly at soft‐hard interfaces of polymer, where smooth transitions have yielded structures that are simultaneously strong and tough.^[^ ^22^ ^]^ Despite these advances, most current methods impose directional constraints on the controllability of spatially distributed material, as they are typically achieved by a layer‐by‐layer variation of materials.^[^ ^26^, ^27^, ^28^ ^]^

Notably, laser powder bed fusion (LPBF) has emerged as a transformative additive manufacturing technology, enabling continuous control at the macro‐scale over material distribution for advanced lattice structure design.^[^ ^29^ ^]^ Recent advances in continuous compositional grading, demonstrated via self‐developed continuous gradient alloy LPBF, have achieved high‐precision fabrication of metallic bulk components with tunable material gradients and geometrically complex interlocking interfaces.^[^ ^30^, ^31^, ^32^ ^]^ These developments have established that spatially controlled material distribution can not only mitigate mechanical stress concentrations but also locally tailor properties such as stiffness and energy absorption.^[^ ^31^ ^]^ However, translating these capabilities into geometrically complex lattice architectures with spatially programmable material distribution remains a formidable engineering challenge. Critically, the interplay between material distribution, mechanical performance, and deformation mechanisms in such multi‐material lattices remains under‐explored, limiting their modeling, prediction, and performance optimization.

In this study, we present a pioneering investigation into the fabrication and mechanical performance of lattice structures with free control over material distribution, produced using a self‐developed LPBF system. The Ti‐Nb system was selected as a demonstrative case, which provides complementary properties: Ti delivers great strength and low density, whereas Nb offers excellent plasticity and toughness. We designed and fabricated two types of lattice structures body‐centered cubic (BCC) truss and Gyroid TPMS) with distinct material distribution configurations, including interface structure (IS) and continuous gradient structure (CGS). The novel AM process allowed precise control over the spatial material distribution, producing intricate lattice geometries with location‐specific properties. The deformation behavior and underlying mechanisms were systematically studied. Statistical analyses elucidated the interactions between material distribution and mechanical response, providing new insights into the role of compositional gradients in enhancing performance. Notably, a strain hardening model was developed to examine the effects of material gradients on the strain hardening behavior. In all, the new multi‐material AM combined with the novel strain hardening model gives access to the design and manufacture of multifunctional devices with tailored material gradients.

## 2 Results and Discussion

### 2.1 Ti‐Nb Gradient‐Material Lattice Structures

**Figure 1:**
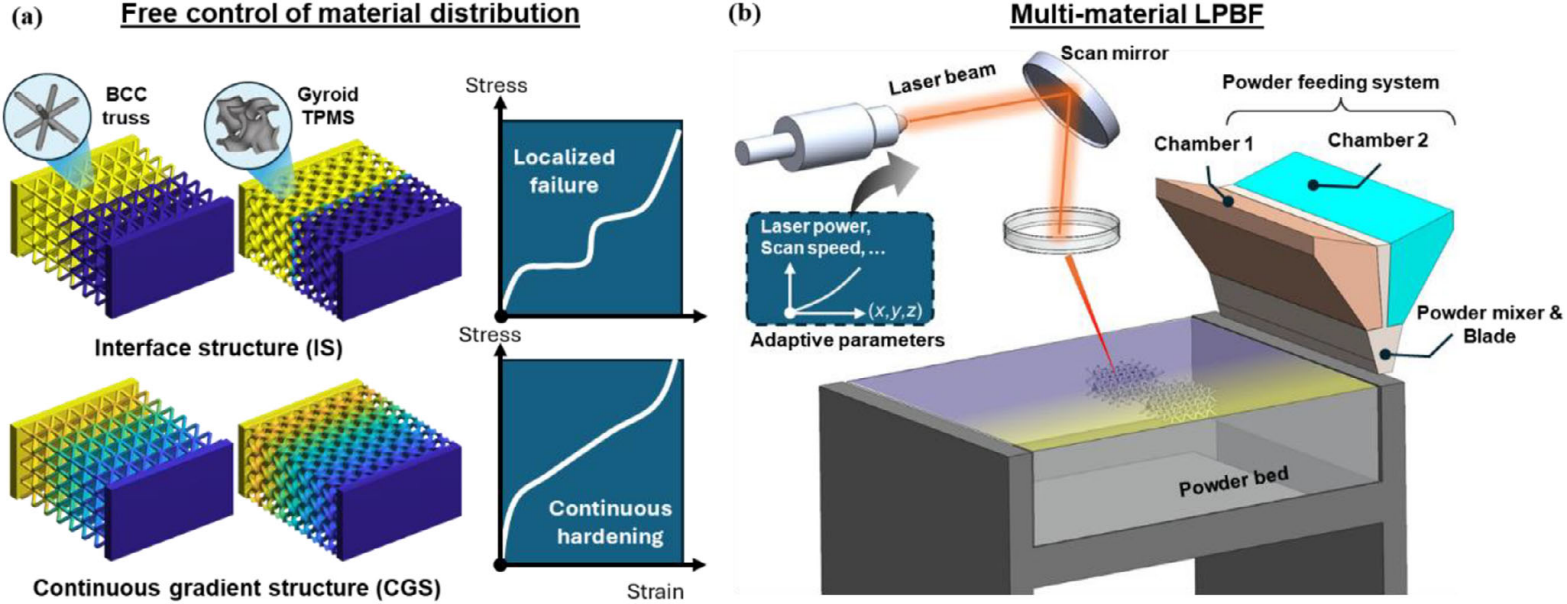
a) Free control of material distribution within lattices; b) Schematic of multi‐material printing via LPBF.

By strategically modulating the material distribution within lattice structures, the compressive deformation response can be tailored to achieve enhanced strain hardening and superior energy absorption. Specifically, the integration of soft and stiff materials enables precise control over the failure sequence within the lattice, thereby guiding the deformation pathway (**Figure** **1**a).

The fabrication of material‐gradient alloy components with distinct lattice architectures via additive manufacturing is shown in Figure 1b. A custom‐designed dual‐chamber powder feeding system enabled precise control over the feed ratios of two base materials, allowing for fine‐tuning of local composition. The mixed powders were uniformly deposited across the powder bed, forming the foundation for high‐resolution, compositionally graded printing. Both IS and CGS structures were produced using an adaptive printing strategy, where key process parameters, including scanning speed and laser power, were adjusted based on the spatial material distribution (Figures S1–S3, Supporting Information). This method ensured the formation of high‐quality, defect‐free transitions, even in the IS structure with its sharp compositional boundary. The full miscibility and ductility of Ti and Nb further minimized interfacial incompatibility, enabling strong metallurgical bonding. Together, enhanced material compatibility and process control improved the structural integrity of both configurations, allowing a focused investigation into the effects of material distribution strategies on mechanical performance, independent of defect‐induced failure.

**Figure 2:**
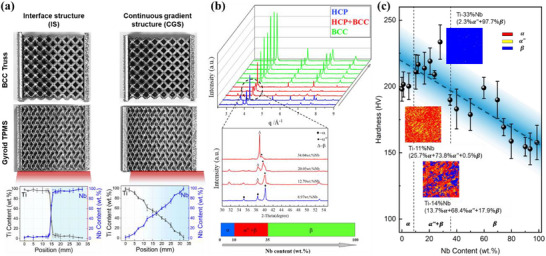
As‐printed lattices with different material configurations and Ti‐Nb alloy properties: a) Ti‐Nb component distribution curve and manufactured samples; b) The phase composition identified by X‐ray diffraction (XRD); c) Hardness distribution with different Nb contents.

The fabrication of Ti‐Nb gradient alloy components via additive manufacturing, featuring distinct lattice structures, is illustrated in **Figure** **2**a. As shown, two distinct material distribution configurations were successfully fabricated, and the experimentally measured compositional profiles align closely with the theoretical design, exhibiting no significant deviations.

In more detail, the interface structure (IS) shows a sharp transition, with the Ti content decreasing from 90% to 10% over a span of 14–15.8 mm, forming a sharp transition zone that constitutes ≈5.6% of the overall length. Specifically, this sharp transition region also maintained good structural integrity, and no cracks or delamination were observed during deformation in both the truss and TPMS lattices (Figure S4, Supporting Information). In contrast, the continuous gradient structure (CGS) exhibits a smooth, uniform variation in composition without abrupt transitions. The mean absolute error of element composition at full gradient range is as low as 3%. Critically, both gradient architectures (IS and CGS) were fabricated without altering the macroscopic geometry or mass of the 3D lattice structures (dimensional and mass data in Table S1, Supporting Information). This confirms that spatially resolved compositional control can be achieved while maintaining near‐identical component mass and volume, underscoring the precision of the multi‐material additive manufacturing process for complex geometries.

To investigate the effect of Nb content on the phase composition in laser‐printed Ti‐Nb alloys, XRD analysis was performed (Figure 2b). Pure Ti exhibits a hexagonal close‐packed (hcp) α phase, while Nb adopts a body‐centered cubic (bcc) β phase. The Ti‐Nb system can be broadly divided into three compositional regions, each corresponding to distinct phase distributions: 1) Low Nb content (0–8.97 wt.%): The alloy remains in the α phase, characterized by its HCP structure. 2) Intermediate Nb content (8.97‐34.04 wt.%): The α′′ phase emerges sharply, while the α phase diminishes. In this range, the alloy exhibits a mixture of α, α′′, and β phases. 3) High Nb content (>34.04 wt.%): As the Nb content increases further, the α′′ phase gradually decreases, and the β phase becomes dominant, leading to a fully β‐phase alloy (Figure S5, Supporting Information). Within the dual‐phase region, a notable shift in the diffraction peak of the α′′ phase (111) toward lower angles is observed, indicating an increase in interplanar spacing with rising Nb content. This shift is attributed to lattice distortion caused by the solid solution of Nb, which expands the lattice.

Micro‐Vickers hardness measurements across the compositional gradient, from pure Ti to pure Nb, are presented in Figure 2c, alongside electron backscatter diffraction (EBSD) results for three key compositions. The hardness of pure Ti is ≈200 HV, while that of pure Nb is ≈155 HV. In the two‐phase region, where Nb content reaches 8.97 wt.%, the hardness increases, reaching a maximum of 232.85 HV at Ti‐28 wt.% Nb. This increase is attributed to two factors: enhanced solid solution strengthening due to Nb addition, and the contribution of the orthorhombic α′′ phase, which acts as a heterogeneous strengthening phase, as illustrated by the EBSD maps.^[^ ^33^ ^]^

Nevertheless, the significant hardness manifests within a narrow compositional range. Beyond this apex, the hardness progressively diminishes with the increasing Nb content, adhering to a linear trend. Consequently, to facilitate the numerical simulation of lattice structures with graded material properties, we applied linear interpolation to estimate the parameters required for the strain hardening law, which describes the mechanical behavior of alloys with varying Nb content. This approach simplifies the modeling process while maintaining accuracy in predicting the mechanical response of the gradient alloy.

### 2.2 Quasi‐Static Compressive Response and Properties

**Figure 3:**
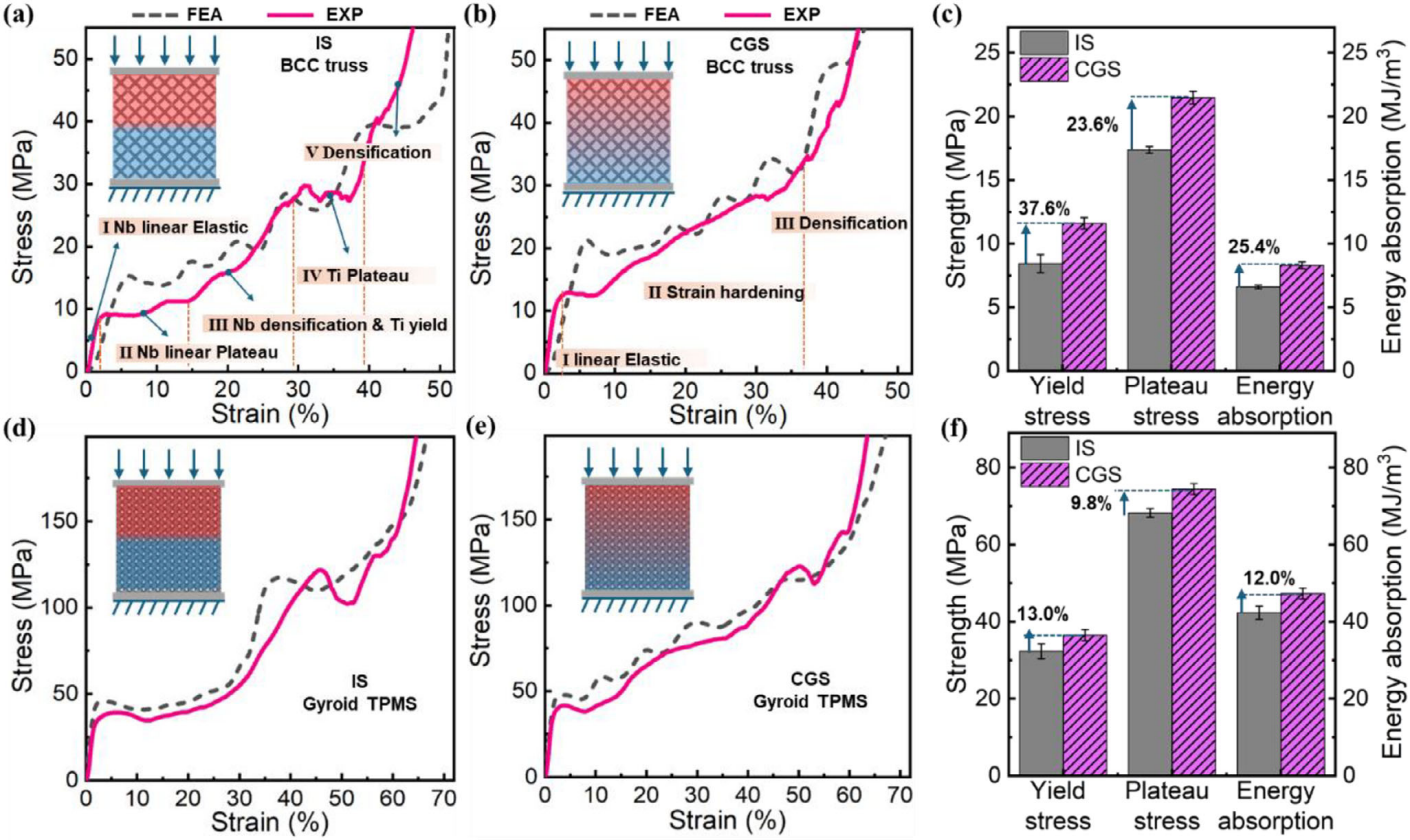
Mechanical responses of lattice structures with different material distributions. Ti‐Nb IS of a) BCC truss and d) TPMS shell, Ti‐Nb CGS of b)BCC truss and e) TPMS shell, strength and energy absorption of c) BCC truss and f) TPMS shell.

**Figure** **3** presents the typical compressive stress versus nominal compressive strain response for two lattice architectures, BCC truss and Gyroid‐type TPMS lattices, under compression. Both lattice types exhibit a similar mechanical response when subjected to material gradients, with the initial deformation and failure zones predominantly occurring in the Nb‐rich regions, which possess a lower elastic modulus and yield strength (Figure 3a,d). The introduction of a continuous material gradient significantly enhances the hardening behavior of both lattice types, with a higher and more consistent strain hardening throughout the deformation process (Figure 3b,e). This enhancement leads to an increase in plateau stress of 23.6% for the truss lattice and 9.8% for the TPMS lattice (Figure 3c,f). Additionally, the energy absorption capacity improves by 25.4% and 12.0%, while the yield stress increases by 37.6% and 13.0% for the truss and TPMS lattices, respectively. Compression tests were conducted at a strain rate of 10^−3^ s^−1^. Under this condition, the direction of loading, whether from Ti to Nb or from Nb to Ti, did not significantly affect the mechanical response of the gradient lattices. This is supported by similar stress‐strain curves and collapse behavior observed (Figure S6, Supporting Information).

In the case of the IS sample, the compressive response (Figure 3a) is characterized by two distinct collapse plateaus, corresponding to the sequential deformation of the Nb and Ti regions. More specifically, the Ti‐Nb IS truss undergoes five distinct stages during compression: an initial linear elastic phase, a Nb collapse plateau, densification of the Nb with an initial yield of Ti, a Ti collapse plateau, and finally, full densification. The first stage reflects the linear elastic deformation of the Nb‐rich portion of the porous Ti‐Nb structure. The second stage, marked by the first collapse plateau, corresponds to the yielding of the Nb regions. The third stage represents a transition, where both densification of the Nb and the early stages of Ti deformation occur simultaneously. The fourth stage, characterized by the second collapse plateau, is associated with the large‐scale plastic deformation of the Ti regions. Finally, the fifth stage signals the densification of the entire Ti‐Nb lattice.

The introduction of a continuous material gradient fundamentally alters the deformation mechanism of both BCC truss and Gyroid TPMS structures. As shown in Figure 3b, the absence of discrete collapse plateaus and the emergence of an ascending stress–strain curve indicates a transition from abrupt, localized failure to a gradient‐driven sequential yielding and strain hardening process. This mechanism initiates in the softer Nb‐rich regions and gradually propagates toward the stiffer Ti‐rich regions, facilitating a layer‐by‐layer deformation mode that enhances both structural stability and energy dissipation capacity. This transition underscores the mechanical benefit of introducing a material gradient, which eliminates abrupt transitions and leads to a more homogeneous and progressive load‐bearing behavior. The underlying mechanism driving this change is the sequential deformation enabled by the gradient design. In CGS structures, the softer material near the top deforms first and begins to harden gradually. As strain increases, the stress is transferred to the stiffer material layers below, which then undergo their own yielding and hardening. This layer‐by‐layer progressive compression is a hallmark of the gradient strategy, effectively distributing the deformation and delaying densification, thereby enhancing both strength and energy absorption capacity.

Although the Gyroid lattice exhibits higher overall strength compared to the BCC truss, the Ti‐Nb IS sample shows a similar five‐stage compressive behavior (Figure 3d). This uniformity in the stress‐strain curve underscores the efficacy of the smooth material gradient in promoting stable, predictable mechanical behavior, in contrast to the more abrupt and concentrated failure modes observed in non‐graded structures (Figure 3e). To further evaluate the benefits of material gradients, we compared the CGS lattices with those made entirely from pure Ti, which possesses higher mechanical strength. The CGS samples demonstrated slightly higher energy absorption capabilities—6.5% and 3.1% improvements for the BCC truss and Gyroid TPMS structures, respectively (Figure S7, Supporting Information). This improvement, achieved alongside a reduced yield strength, highlights the potential of material gradient strategies for energy‐absorbing applications. Additionally, the integration of Ti and Nb offers functional advantages, such as combining lightweight properties with high‐temperature resistance due to Nb's elevated melting point.

### 2.3 Compressive Deformation Behavior

**Figure 4:**
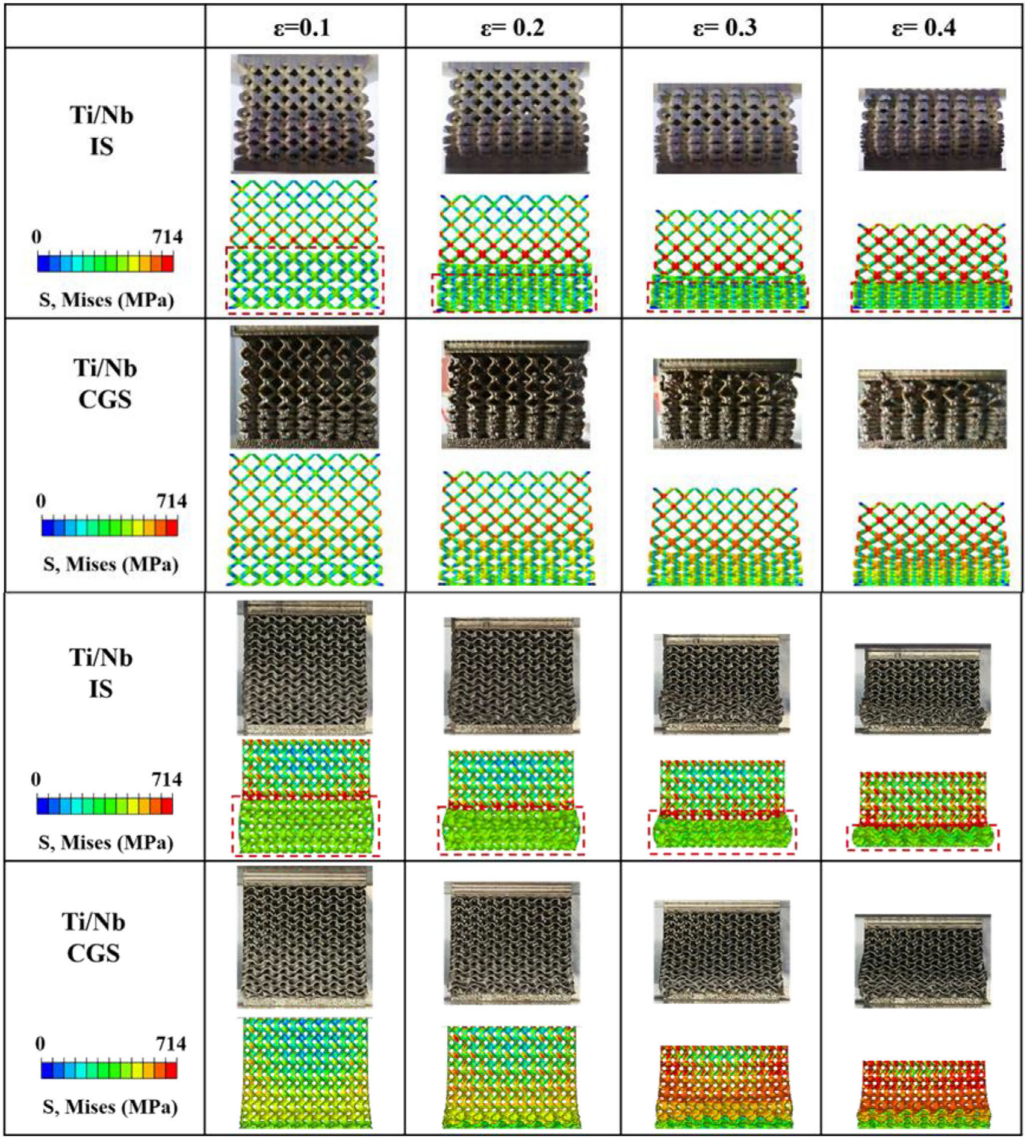
Compression behaviors of Ti‐Nb BCC Truss and TPMS lattice structures.

To further investigate the mechanical response of lattices with a material gradient, we conducted analogous tests using numerical simulation. The graded lattice structures were modeled using a composition‐dependent strain hardening law, where material parameters were interpolated based on the local composition (Figure S8, and Tables S2 and S3, Supporting Information). The structures were discretized into segments, each assigned corresponding mechanical properties, allowing the finite element model to capture the spatially varying plastic response of the gradient alloy. As illustrated in Figure 3, the outcomes from the finite element simulation agree well with the experimental data. **Figure** **4** presents a comparison of deformation behavior for the BCC truss and Gyroid TPMS lattices, respectively. The deformation patterns observed experimentally and predicted by FE models match very well, providing good validation of the numerical models. Notably, the material gradients induce a transition in the deformation mechanisms for both lattice types.

Interestingly, in contrast to conventional single‐material lattices^[^ ^15^, ^34^ ^]^ both BCC lattices and TPMS structures with a smooth material gradient demonstrate a progressive collapse behavior with regulated plastic propagation. It has been established that global shear bands result in significant fluctuations in stress‐strain curves, hence compromising energy absorption. While certain geometric designs can partially alleviate the nucleation and propagation of shear bands, the large‐scale failure bands remain inadequately mitigated. Comparatively, the material gradient can significantly influence the deformation modes of lattices.

In the case of BCC lattice structures, Figure 4 illustrates the compressive behavior of two samples with two compositional configurations. For the IS sample, significant stress concentrations were observed at the truss nodes on the Ti side, with large plastic deformations predominantly localized on the Nb side. Examination of the equivalent plastic strain (ε_eq_) for the IS sample revealed that plastic deformation primarily occurred on the Nb side under compressive strain up to 30%. The Ti side, in contrast, retained its original shape until the densification of the Nb side, after which yielding commenced in the Ti lattice. This single‐layer stress concentration accelerates failure in the IS sample. In contrast, the CGS structure demonstrates a spatially regulated plastic progression from soft to stiff regions, enabled by the continuous variation in local yield strength. This results in a layer‐by‐layer collapse mode, which not only mitigates stress localization but also promotes a more controlled and predictable deformation behavior.

Similarly, the compressive behavior of Ti‐Nb TPMS lattices is depicted in Figure 4. In the Ti‐Nb IS sample, deformation initiated across the entire Nb region, whereas in the Ti‐Nb CGS sample, failure began in the lower‐strength layers, progressing systematically from the Nb layer to the Ti layer in a gradual, layer‐by‐layer fashion. This difference in deformation behavior is reflected in the shape of the lattice under compression. At 20% compressive strain, the IS sample exhibited symmetrical outward bulging on both sides, indicative of plastic deformation predominantly occurring in the Nb section. In contrast, the CGS sample showed the widest edge at the lower boundary, with the width increasing progressively along the gradient direction.

Regarding stress concentration, the TPMS lattice exhibited behavior consistent with the BCC lattice. The IS sample showed pronounced stress concentration near the Ti side, particularly at the interface, while the CGS sample displayed a more gradual increase in stress along the gradient direction, effectively alleviating localized stress concentrations.

### 2.4 The Energy Absorption Capability

Lattice structures are widely employed in energy absorption applications, ranging from protective devices to packaging solutions. By manipulating geometric parameters and material configurations, structures with tunable strength and optimized energy absorption capabilities can be designed. A quantitative assessment of energy absorption is crucial for selecting appropriate lattice candidates for these applications. Energy absorption properties are intrinsically linked to the collapse observed during compressive loading, which can be quantified through the numerical integration of the compressive stress‐strain response. This is expressed as:^[^ ^35^ ^]^

(1) $$W=\int_{0}^{\varepsilon_{D}}\sigma \left(\varepsilon \right)d\varepsilon \;$$

(2) $$\eta \;\left(\varepsilon \right)=\frac{1}{\sigma \left(\varepsilon \right)}\;\int_{0}^{\varepsilon }\sigma \left(\varepsilon \right)d\varepsilon$$

where σ(ε) is the compressive stress corresponding to the compressive strain of ε. The ε_*d*_ represents the densification strain, defined as the strain at which the mechanical energy absorption efficiency reaches its global maximum value (Figure S9, Supporting Information). This definition ensures that all plateau regions contributing to energy absorption, including secondary plateaus, are fully captured.

The specific energy absorption (SEA)^[^ ^36^ ^]^ is the energy absorption per unit mass, and the expression is obtained as follows:

(3) $$SEA=\frac{W}{\rho }$$

where *W* is the total energy absorption, ρ is the density of the sample.

The energy absorption capacities of the IS and CGS BCC truss structures are 6.62 and 8.30 MJ m^−^^3^, respectively, while those of the IS and CGS TPMS structures are significantly higher, at 42.31 and 47.29 MJ m^−^^3^, respectively. Notably, the structures incorporating material gradients (CGS) demonstrate superior energy absorption performance compared to their IS counterparts. Specifically, CGS truss structures exhibit enhancements of 25.4% in energy absorption and 21.7% in SEA, while CGS TPMS structures show improvements of 12.0% and 13.8% in energy absorption and SEA, respectively.

The superior energy absorption of CGS lattices is intrinsically linked to the gradient‐controlled deformation pathway. The failure initiates in the Nb‐rich region with the lowest yield strength and is followed by a gradual activation of stronger layers. This behavior results in cumulative hardening and delayed densification, which collectively enhance load‐bearing capacity and energy absorption efficiency. This gradient‐induced distributed plasticity stands in stark contrast to the abrupt collapse observed in IS or monolithic lattices, where localized deformation limits post‐yield performance. This behavior contrasts with the response of non‐graded lattices, where deformation tends to localize in the weakest regions without subsequent hardening, leading to reduced load‐bearing capability after initial yielding.

In order to gain a deeper understanding of the mechanical energy absorption property, power functions were utilized in order to fit the cumulative energy absorption curves as a function of strain.^[^ ^37^ ^]^ The fitted exponents for the CGS BCC truss and G‐type TPMS structures, as shown in Figure S10 (Supporting Information), indicate the rate of increase in cumulative energy absorption throughout the compression process. Notably, the exponents for the CGS samples are higher than those for the IS samples, reflecting their superior energy absorption performance.

In conclusion, the results demonstrate that material gradients significantly enhance the energy absorption capacity of lattice structures, making them promising candidates for energy absorption applications.

### 2.5 Material Gradient‐Enabled Strengthening Mechanism

One of the key characteristics of lattice intertwining is that large deformations cause beam or shell members to come into contact, thereby enhancing both the stiffness and strain energy density of the structure. Simultaneously, the constitutive materials may contribute to this hardening effect, wherein regions experiencing yield undergo larger plastic strains accompanied by increased strength. Consequently, lattice structures collapse in a manner analogous to the strain hardening observed in bulk materials. In this study, the distribution of plastic strain along the compressive directions of lattice structures was extracted and subjected to statistical analysis.

**Figure 5:**
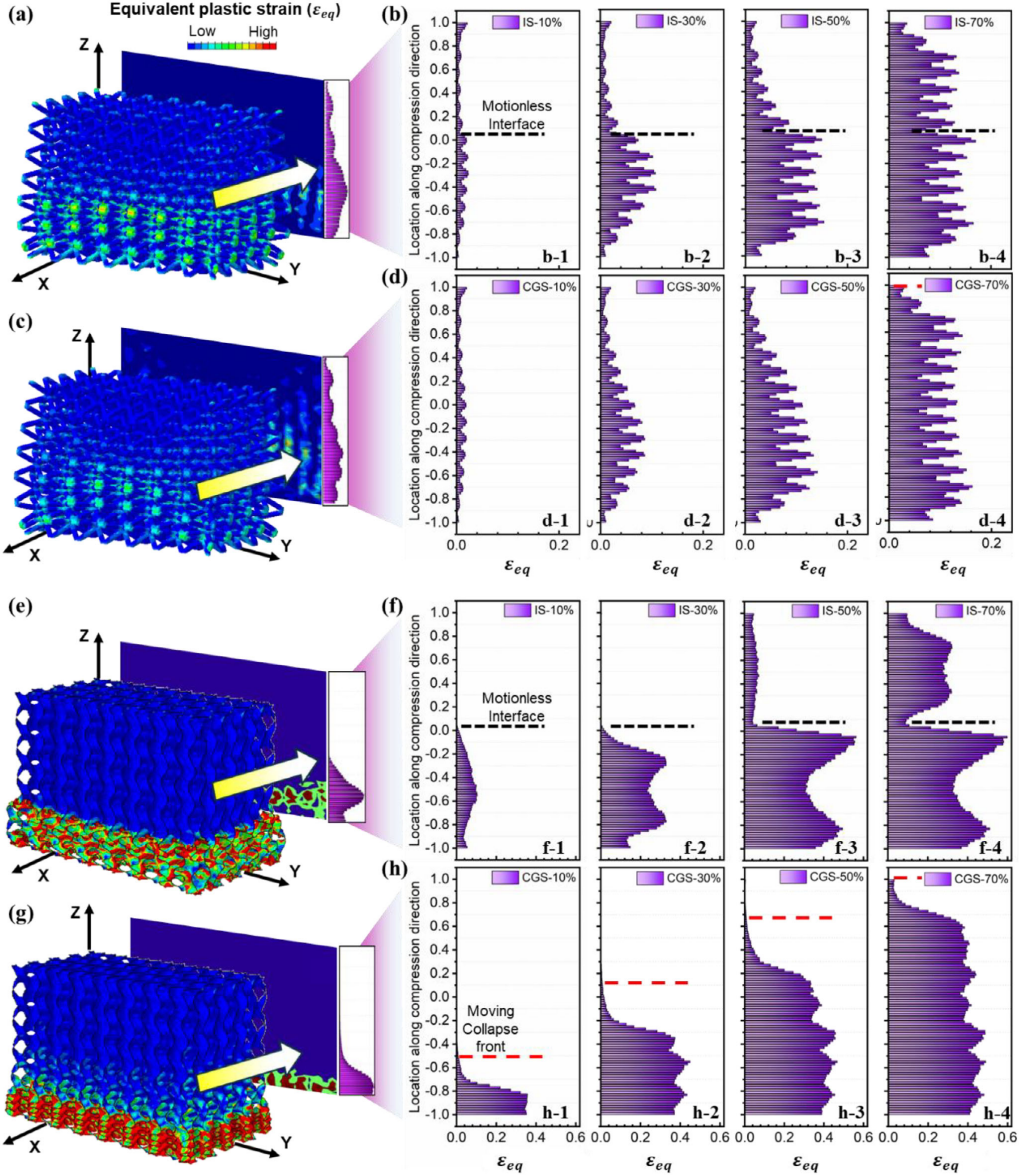
Plastic strain distribution of BCC truss and G‐type TPMS during compressive progress: a,b) distribution of ε_*eq*_ of the IS truss; c,d) distribution of ε_**eq**_ of the CGS truss; e,f) distribution of ε_**eq**_ of IS TPMS; g,h) distribution of ε_**eq**_ of CGS TPMS.

As depicted in **Figure** **5**, equivalent plastic strain (ε_eq_) distributions along the compression axis were plotted with respect to different stages of large deformation. For the IS sample, a stationary interface, as highlighted in Figure 5a,e, is observed. At moderate compressive strains, plastic deformation is primarily concentrated on the softer side, composed of Nb material. In the densification stage, the ε_eq_ distribution becomes progressively more uniform along the compression direction. In contrast, CGS lattices exhibit a continuous plastic strain gradient along the loading direction, without any stationary interface (Figure 5b). The progressive collapse initiates in the compliance‐dominated Nb‐rich region and advances toward the Ti‐rich region in a regulated manner. This observation provides direct evidence for the gradient‐induced sequential plasticity, confirming that compositional gradients can serve as a governing factor in orchestrating the deformation front and suppressing shear localization.

These material gradients effectively regulate the propagation of plastic regions and distribute the plastic strain across multiple layers. Consequently, inhibition of extensive global shear band development can be accomplished, thereby improving structural integrity and energy absorption.

The disparity in ε_eq_ distribution becomes more pronounced in Gyroid TPMS lattices. As illustrated in Figure 5e–h, the TPMS structure displays a more significant response to variations in material distribution. Particularly, the IS sample reveals a clear stationary interface in the ε_eq_ distribution during the deformation process. During moderate compressive strains, the soft material accounts for nearly all plastic deformation (>99% for a strain of 30%). The introduction of a material gradient fundamentally reconfigures the collapse behavior of TPMS structures. As shown in Figure 5e,g, the CGS sample exhibits a more uniform and elevated distribution of equivalent plastic strain, indicative of a broader, more delocalized plastic response. The spatially propagating plastic front guarantees that a large volume of the material undergoes plastic deformation in a temporally staggered manner, thereby maintaining a high and steady flow stress throughout compression. This redistribution is further captured in the statistical analysis (Figure 5h), where the regular spacing of ε_eq_ frequency peaks reflect a well‐coordinated, layer‐by‐layer folding process. These observations reveal that the gradient not only modifies local mechanical properties, but also actively programs the global deformation pathway—shifting the failure mode from abrupt, interface‐driven collapse to a continuous, gradient‐guided plastic evolution.

Furthermore, the magnitude of ε_eq_ in the Gyroid lattice is substantially larger than that in the BCC truss. This discrepancy is primarily attributed to the bending‐to‐stretching ratio during deformation.^[^ ^38^ ^]^ Specifically, the BCC truss is a bending‐dominated lattice, where plastic deformation is primarily localized at the truss joints. Conversely, Gyroid TPMS structures are stretching‐dominated, which imparts greater stability at comparable relative densities. Thus, the IS sample, the TPMS lattice exhibits an extended plateau in the deformation stage, whereas the BCC lattice shows a shorter plateau, followed by gradual hardening. Moreover, in the stretching‐dominated TPMS, the material is more likely to experience in‐plane strain, contributing to higher plastic deformation and thereby a greater magnitude of ε_eq_.

The strain hardening of lattice structures is closely associated with their mechanical stability and energy absorption capacity during large strain deformations. Strain hardening, particularly the stress‐strain behavior in the plateau and densification stages, can be described using both linear and nonlinear hardening laws. In this study, we employed an analytical strain hardening model as proposed by:^[^ ^39^ ^]^

(4) $$\sigma =\sigma_{p}+\gamma \frac{e}{e_{D}}+\alpha Ln\left(\frac{1}{1-{\left(e/e_{D}\right)}^{\beta }}\right)$$

where the σ_*p*_ denotes the initial level of the plateau region, which is the compressive strength of the lattice structures immediately after the initial plastic yielding. The second term of the model describes linear strain hardening, where the slope of the stress‐strain curve is defined by the linear hardening coefficient, γ. The third term addresses nonlinear strain hardening, characterized by a scale factor, α, and shape factor β.

Herein, the lattices with graded materials were fitted using the strain hardening model. The data extracted from stress‐strain curves at the plateau collapse and densification stages were used to evaluate the parameters via Genetic Algorithm (GA). Figure S11 (Supporting Information) presents the stress‐strain curves for both the CGS truss and CGS TPMS lattice structures. The results demonstrate that this model effectively captures the compaction stress behavior during the plastic collapse of these structures. The parameter γ represents the initial linear strain hardening coefficient, primarily influenced by the deformation mode of the lattice elements (e.g., bending or stretching). Stretching‐dominated structure, Gyroid TPMS, exhibits higher γ values due to its enhanced load‐bearing capacity during early deformation (Figure S12a, Supporting Information). The parameter α corresponds to the plateau stress following the elastic regime and marks the onset of structural collapse. The Gyroid TPMS also shows a significantly higher α than the bending‐dominated BCC truss. The parameter β governs the nonlinear hardening behavior in the densification stage, where geometric contact and material compaction dominate. The BCC truss shows a higher β due to beam‐to‐beam contact and subsequent material hardening, while TPMS structures, with more deformable and open curved surfaces, exhibit lower β values and more gradual densification (Figure S12b, Supporting Information). These parameter values are summarized in Table S4 (Supporting Information), with statistical comparisons highlighting the influence of geometry on plastic deformation mechanisms.

**Figure 6:**
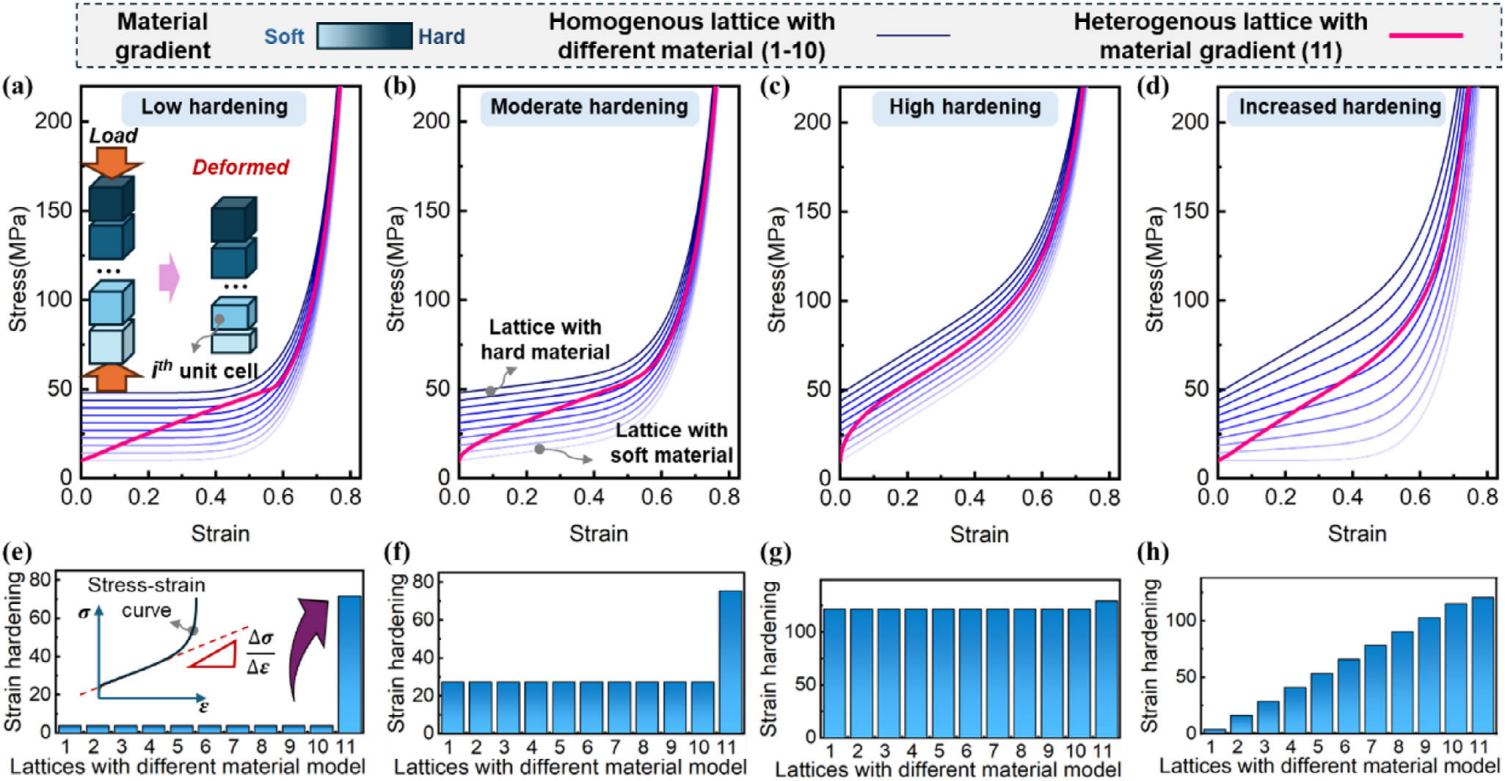
Material gradient enabled strain hardening effect under four material cases. The stress‐strain curves and strain hardening rate: a,e) Increased yield strength with low hardening rate, b,f) Increased yield strength with moderate hardening rate; c,g) Increased yield strength with high hardening rate; d,h) Both increased yield strength and hardening rate.

We further utilized this model to investigate how material gradient influences the mechanical response of architected lattices. The IS samples exhibit two distinct compressive plateaus, corresponding to the plastic collapse of lattices composed of single materials. This observation highlights the intrinsically low strain hardening behavior of both constituent materials of Ti and Nb. The deformation response of the lattices is governed not only by the material distribution configurations but also by the constitutive properties of materials. Thus, four distinct cases were examined: increased yield strength with low, moderate, and high constant strain hardening rates, as well as a combination of increased yield strength and hardening rate (**Figure** **6**). By adjusting the initial yield stress, σ_*p*_, and the strain hardening coefficient, γ, we generated various stress‐strain curves, corresponding to different constitutive materials. Specifically, σ_*p*_ was varied from 10 to 50 MPa, while γ ranged from 0 to 100 MPa.

The resulting stress‐strain curves for homogeneous lattices with varying material properties are calculated based on Equation (4). For heterogeneous lattices with graded materials, the stress‐strain response was computed based on stress equilibrium. To simplify the calculations, we assumed each unit cell to be homogeneous, with its compressive strain determined by Equation (4) under a given load. The overall compressive strain of the lattice was then evaluated by summing the strains of the individual unit cells, as schematically illustrated in Figure 6.

(5) $$\bar{\varepsilon }=\frac{\Delta V}{V_{0}}=\frac{\sum \Delta V_{i}}{V_{0}}=\frac{\sum \Delta V_{i,0}\varepsilon_{i}}{V_{0}}$$

where *V*_0_ is the initial overall volume of the lattice structure, Δ*V_i_* is the volume change of *i^th^* unit cell during deformation, Δ*V*_*i*,0_ is the initial volume of unit cells, and ε_*i*_ is the strain of *i^th^* cell.

It has been demonstrated that the mechanical response of a heterogeneous lattice is strongly influenced by the variation of materials. The results reveal that material gradients enhance the strain hardening effect across all four cases. Herein, we define the $\frac{\Delta \sigma }{\Delta \varepsilon \;}$ as the strain hardening value for the lattice structures. This effect is particularly pronounced in materials with low hardening rates. For lattices with σ_*p*_ values ranging from 10 to 50 MPa and a zero linear hardening coefficient (γ = 0), the strain hardening in heterogeneous lattices increased ≈17‐fold compared to homogeneous ones, from 3.8 to 68.8. In materials with increased yield strength and high hardening rates, the heterogeneous lattices exhibited substantial strain hardening, with increases of more than 30‐fold relative to lattices composed of the softest materials. Comparatively, for materials with initially high hardening rates, the enhancement in strain hardening was less pronounced.

These findings demonstrate that material gradients can impart a pronounced strain hardening effect, even in constitutive materials with inherently low strain hardening rates. Analytical predictions exhibit strong agreement with experimental observations, revealing that yielding initiates in the weakest lattice layer and sequentially propagates to stronger layers. This behavior is consistent with a layer‐by‐layer collapse mechanism, notably occurring in the absence of shear band formation. The robust confinement provided by the material gradient effectively stabilizes plastic flow, promoting a controlled deformation mode and exceptional energy absorption capacity.

### 2.6 Further Discussion

**Figure 7:**
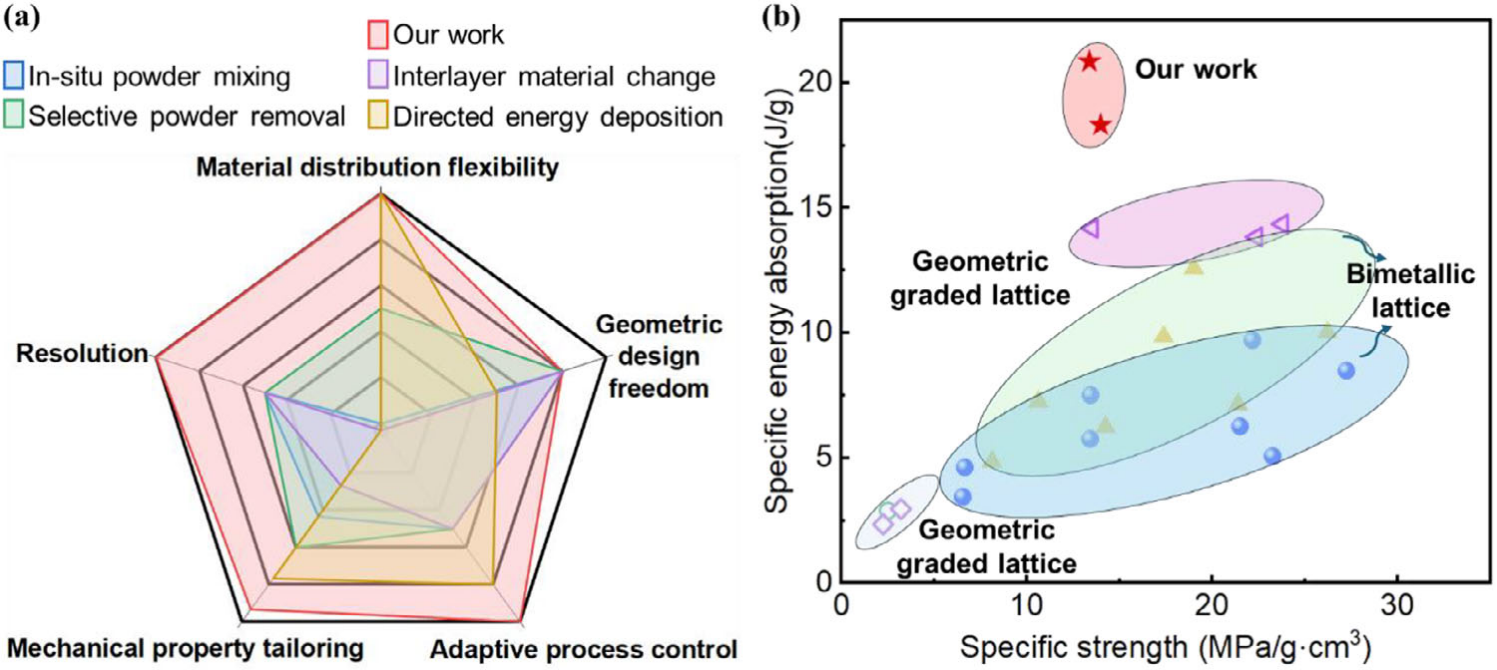
Comparative analysis: a) Radar chart illustrating the feature‐level comparison between the proposed method and other major classes of multi‐metallic material printing technologies,^[^ ^25^, ^27^, ^28^, ^29^, ^31^ ^]^ b) plot of specific energy absorption versus specific strength, comparing the proposed method with reported data from bimetallic lattice^[^ ^28^, ^40^ ^]^ geometric graded lattice.^[^ ^41^, ^42^, ^43^ ^]^

A comprehensive evaluation of current multi‐metallic material printing technologies is presented via a radar chart (**Figure** **7**a), benchmarking key performance metrics relevant to advanced component design and fabrication. These metrics include material distribution flexibility, geometric design freedom, adaptive process control, mechanical property tailoring, and spatial resolution. For material distribution flexibility, we consider the ease of fabrication, spatial control over composition, and the diversity of achievable gradients. Comparative performance across these criteria is detailed in Table S5 (Supporting Information), with our method emerging as the only approach that achieves high scores across all five axes.

We introduce a novel LPBF strategy that enables continuous material gradients within architected lattice structures via a single‐step, parameter‐adaptive process. In contrast to conventional multi‐material LPBF, which typically relies on sequential deposition and multiple fabrication stages, our approach enables adaptive process parameters according to local composition. This enables simultaneous printing of Ti and Nb, two metals with markedly different melting points (Ti: 1668 °C; Nb: 2468 °C), without requiring intermediate processing steps.

Existing approaches offer limited gradient control: in situ powder mixing or interlayer composition changes are constrained to 1D gradients, while selective powder removal systems enable 3D material placement but only in discrete patches, precluding smoothly varying transitions. Directed energy deposition provides full 3D compositional control but suffers from limited resolution and struggles with the fine features required in lattice architectures.

Our method enables high‐resolution control over material composition, achieving sharp transitions within 2 mm (≈5.6% of the total sample length) and smooth gradient distributions with compositional deviations kept below 3%. These precisely engineered gradients have a significant impact on mechanical performance. By employing a composition‐dependent strain hardening model, we accurately predict and experimentally validate the deformation behavior of the graded lattice structures. The presence of compositional gradients facilitates the formation of a progressive collapse front, initiating in softer regions and advancing toward stiffer areas. This controlled, layer‐by‐layer failure mechanism results in enhanced energy absorption when compared to both geometrically graded and bimetallic lattice counterparts, as shown in Figure 7b.

While our approach provides significant advantages in design flexibility, structural performance, and manufacturing efficiency, it also presents certain limitations. One key challenge is powder sustainability, as mixed powders generated during printing are difficult to recycle or reuse without complex separation processes. In addition, the expanded design space involving both geometry and material composition introduces substantial complexity in process optimization. This requires careful calibration of material compatibility, thermal behavior, and microstructural evolution, often demanding extensive experimental effort. To overcome these challenges and fully realize the potential of functionally graded materials, future research can incorporate AI‐driven tools for inverse design, process parameter prediction, and multi‐objective optimization. The integration of machine learning into our parameter‐adaptive LPBF framework could accelerate material selection, reduce trial‐and‐error experimentation, and enable the discovery of high‐performance, sustainable architectures.

## 3 Conclusion

The capacity to spatially program material composition within architected lattice structures represents a significant advancement in the design of high‐performance, multi‐material systems. In this work, we demonstrate the fabrication of truss‐ and TPMS‐based lattices with tailored compositional gradients using a custom‐developed laser powder bed fusion (LPBF) platform. This constitutes the first successful realization of spatially resolved material gradients within these classic lattice topologies without altering the overall mass, volume, or geometry of the structures. Two distinct material distribution strategies, sharp interfaces and continuous gradients, were systematically investigated. The continuous gradient strategy resulted in markedly enhanced mechanical performance, with improvements in plateau stress (up to 23.6% in BCC lattices and 9.8% in Gyroid structures) and energy absorption (25% and 12%, respectively) relative to their sharp‐interface counterparts. These enhancements arise from a compositionally programmed deformation mechanism, where a plastic front propagates from soft to stiff regions. This stabilizes plastic flow, suppresses strain localization, and enhances strain hardening, highlighting material gradients as a powerful design tool to program deformation and optimize performance without changing structural geometry. Finite element modeling and a strain hardening‐based constitutive framework further elucidated the role of compositional gradients in governing global deformation behavior. Notably, even in systems composed of materials with inherently limited hardening capacity, graded distributions imparted a pronounced enhancement in strain hardening response. These findings establish compositional grading as a powerful mechanism to modulate and optimize the mechanical performance of architected materials. The integration of such gradients offers a promising route toward the development of next‐generation lattice structures with tunable, site‐specific functions.

## 4 Experimental Section

### LPBF Process and Fabrication of Multi‐Material Lattices

The LPBF experiments were conducted on a self‐developed multi‐material printing system, equipped with a 400 W fiber laser (Figure 1a). The laser spot size was maintained at 30 µm throughout the process. A custom‐designed powder feeding system with dual chambers allowed precise control over the feeding ratio of two materials, enabling the fine‐tuning of element compositions. The mixed powders were then uniformly spread across the powder bed for printing. Detailed information on the gradient powder feed setup is available in our previous work.^[^ ^44^ ^]^ To demonstrate the effectiveness of material gradients, two representative lattice structures were selected: a BCC truss and a Gyroid‐type TPMS, as illustrated in Figure 1b. These structures, composed of strut and shell elements, are commonly used in lattice‐based lightweight design. The BCC truss was modeled using SolidWorks 2024, while the Gyroid TPMS was generated in MATLAB based on implicit functions.^[^ ^45^ ^]^ To optimize printing accuracy, the wall thickness of the Gyroid shell was set to 200 µm, and the truss diameter of the BCC lattice was 560 µm. The cell size was set as 3.75 mm. Two types of material gradient profiles, including interface and continuous gradient, were implemented.

Gas‐atomized spherical powders of commercially pure niobium (Nb) and titanium (Ti) were used as feedstock for the LPBF process. The particle size distribution of the powders ranged from 13 to 53 µm, as shown in Figure S1 (Supporting Information). The LPBF process parameters, including laser power and scan speed, were adaptively adjusted based on the alloy composition (Figures S2 and S3, Supporting Information). A layer thickness of 40 µm and a hatching distance of 80 µm were maintained during printing, with a 67° rotation between successive layers of the laser scanning path.

### Characterization

Following fabrication, the printed samples were separated from the build plate using wire electrical discharge machining (EDM). No post‐processing heat treatments were applied. The morphology and chemical composition of the as‐printed samples were examined using a scanning electron microscope (SEM, Hitachi FE‐SEM SU8000) equipped with energy‐dispersive X‐ray spectroscopy (EDS). Hardness measurements were taken using an EM‐1500L hardness tester, with each reported value representing an average of five measurements. Indentations were executed with a load of 150 g and a dwell time of 10 s. XRD analysis was performed using a Rigaku Ultima‐IV diffractometer, with a 2θ scan range of 20°–90° and a scanning speed of 5°/min. Microstructural analysis was conducted using an EBSD system (OXFORD Symmetry S2). The mechanical properties of the constituent materials were evaluated through uniaxial tensile tests on dogbone specimens according to ASTM E8 standard. To investigate the mechanical performance and energy absorption of the lattice structures, quasi‐static compression tests were carried out using a WDW‐200D universal testing machine. The tests were conducted at a constant strain rate of 10^−3^ s^−1^. The reliability and reproducibility of the experimental results were validated through statistical analysis of three repeated tests. The results demonstrated consistent behavior across samples, confirming good repeatability (Figure S13, Supporting Information).

### Finite Element Analysis

The multi‐material lattices' deformation behavior was simulated using the finite element method (FEM) in ABAQUS to elucidate the experimental data. BCC truss and TPMS shell lattice structures were discretized using C3D4 and S3 elements, respectively. A convergence analysis was performed to ensure the precision of the numerical results, with element sizes of 0.2 mm used for C3D4 and S3 elements. The boundary conditions for the uniaxial compression tests were simulated by constraining the lattice structures between two rigid shell plates. The bottom plate was fully fixed, while the upper plate was displaced downward at a constant speed. All simulations were conducted at room temperature. Strain hardening plays a critical role in determining the mechanical stability of lattice structures under plastic deformation, particularly after the initial yield. To simulate the mechanical response of graded lattice structures, a simplified strain hardening law was adopted:^[^ ^46^, ^47^ ^]^

(6) $$\sigma_{s}=A\left(x\right)+B\left(x\right){\varepsilon_{e}}^{n\left(x\right)}$$

where *x* denotes the Nb atomic ratio, and the parameters *A*(*x*), *B*(*x*), and *n*(*x*) were composition‐dependent and calibrated using tensile tests on six different Ti–Nb alloy compositions. These parameters exhibited linear or quadratic relationships with elemental composition, enabling interpolation across the gradient (Figure S8, and Tables S2 and S3, Supporting Information). The graded lattices were discretized into segments, each assigned composition‐specific material parameters. This method allows the FEA model to capture the spatially varying plastic behavior of the gradient alloys while maintaining computational efficiency.

## Conflict of Interest

The authors declare no conflict of interest.

## Supporting information



Supporting Information

## Data Availability

The data that support the findings of this study are available from the corresponding author upon reasonable request.

## References

[1] D. Nepal , S. Kang , K. M. Adstedt , K. Kanhaiya , M. R. Bockstaller , L. C. Brinson , M. J. Buehler , P. V. Coveney , K. Dayal , J. A. El‐Awady , Nat. Mater. 2023, 22, 18. 36446962 10.1038/s41563-022-01384-1

[2] G. L. Koons , M. Diba , A. G. Mikos , Nat. Rev. Mater. 2020, 5, 584.

[3] H. Dai , W. Dai , Z. Hu , W. Zhang , G. Zhang , R. Guo , Adv. Sci. 2023, 10, 2207192. 10.1002/advs.202207192 PMC10190572 36935371

[4] M. Zhang , N. Zhao , Q. Yu , Z. Liu , R. Qu , J. Zhang , S. Li , D. Ren , F. Berto , Z. Zhang , Nat. Commun. 2022, 13, 3247. 35668100 10.1038/s41467-022-30873-9 PMC9170714

[5] A. J. D. Shaikeea , H. Cui , M. O'Masta , X. R. Zheng , V. S. Deshpande , Nat. Mater. 2022, 21, 297. 35132213 10.1038/s41563-021-01182-1

[6] Z. Gao , X. Zhang , Y. Wu , M.‐S. Pham , Y. Lu , C. Xia , H. Wang , H. Wang , Nat. Commun. 2024, 15, 7373. 39191786 10.1038/s41467-024-51757-0 PMC11349770

[7] J. Ding , Q. Ma , X. Li , L. Zhang , H. Yang , S. Qu , M. Y. Wang , W. Zhai , H. Gao , X. Song , Adv. Sci. 2024, 11, 2402727. 10.1002/advs.202402727 PMC11538692 39285656

[8] Y. Wang , X. Zhang , Z. Li , H. Gao , X. Li , Proc. Natl. Acad. Sci 2022, 119, 2119536119. 10.1073/pnas.2119536119 PMC9407660 35969756

[9] W. Liu , S. Janbaz , D. Dykstra , B. Ennis , C. Coulais , Nature 2024, 634, 842. 39415014 10.1038/s41586-024-08037-0

[10] P. Jiao , J. Mueller , J. R. Raney , X. Zheng , A. H. Alavi , Nat. Commun. 2023, 14, 6004. 37752150 10.1038/s41467-023-41679-8 PMC10522661

[11] X. Zhang , L. Jiang , X. Yan , Z. Wang , X. Li , G. Fang , Mater. Des. 2023, 226, 111564.

[12] L. Yan , Y. Chen , F. Liou , Addit. Manuf. 2020, 31, 100901.

[13] M. Zhao , B. Ji , D. Z. Zhang , H. Li , H. Zhou , Addit. Manuf. 2022, 52, 102676.

[14] M. Zhao , D. Z. Zhang , F. Liu , Z. Li , Z. Ma , Z. Ren , Int. J. Mech. Sci. 2020, 167, 105262.

[15] Y. Tian , X. Zhang , B. Hou , A. Jarlöv , C. Du , K. Zhou , Proc. Natl. Acad. Sci. 2024, 121, 2407362121. 10.1073/pnas.2407362121 PMC11513908 39401355

[16] J. Ding , S. Qu , L. Zhang , M. Y. Wang , X. Song , Addit. Manuf. 2022, 58, 103061.

[17] M. Rafiee , R. D. Farahani , D. Therriault , Adv. Sci. 2020, 7, 1902307. 10.1002/advs.201902307 PMC7312457 32596102

[18] J. Cheng , R. Wang , Z. Sun , Q. Liu , X. He , H. Li , H. Ye , X. Yang , X. Wei , Z. Li , Nat. Commun. 2022, 13, 7931. 36566233 10.1038/s41467-022-35622-6 PMC9789974

[19] S.‐J. Ahn , H. Lee , K.‐J. Cho , Nat. Commun. 2024, 15, 3605. 38714684 10.1038/s41467-024-47480-5 PMC11076495

[20] B. Zheng , Y. Xie , S. Xu , A. C. Meng , S. Wang , Y. Wu , S. Yang , C. Wan , G. Huang , J. M. Tour , Nat. Commun. 2024, 15, 4541. 38806541 10.1038/s41467-024-48919-5 PMC11133382

[21] D. Gu , X. Shi , R. Poprawe , D. L. Bourell , R. Setchi , J. Zhu , Science 2021, 372, abg1487. 10.1126/science.abg1487 34045326

[22] M. Saldívar , E. Tay , A. Isaakidou , V. Moosabeiki , L. Fratila‐Apachitei , E. Doubrovski , M. J. Mirzaali , A. Zadpoor , Nat. Commun. 2023, 14, 7919. 38086804 10.1038/s41467-023-43422-9 PMC10716482

[23] S. M. Sajadi , L. Vásárhelyi , R. Mousavi , A. H. Rahmati , Z. Kónya , Á. Kukovecz , T. Arif , T. Filleter , R. Vajtai , P. Boul , Sci. Adv. 2021, 7, abc5028. 10.1126/sciadv.abc5028 PMC8262818 34233870

[24] J. Bauer , M. Sala‐Casanovas , M. Amiri , L. Valdevit , Sci. Adv. 2022, 8, abo3080. 10.1126/sciadv.abo3080 PMC9385151 35977008

[25] L. Liu , D. Wang , G. Deng , C. Han , Y. Yang , J. Chen , X. Chen , Y. Liu , Y. Bai , J. Manuf. Process. 2022, 82, 51.

[26] A. Nazir , O. Gokcekaya , K. M. M. Billah , O. Ertugrul , J. Jiang , J. Sun , S. Hussain , Mater. Des. 2023, 226, 111661.

[27] L. Liu , D. Wang , C. Han , Y. Li , T. Wang , Y. Wei , W. Zhou , M. Yan , Y. Liu , S. Wei , J. Alloys Compd. 2024, 978, 173508.

[28] M. Zhang , Y. Yang , D. Wang , C. Song , J. Chen , Mater. Des. 2019, 165, 107583.

[29] D. Wang , L. Liu , J. Tang , Y. Liu , C. Wei , Z. Weng , J. Shao , H. Tan , W. Zhou , B. Neirinck , Int. J. Extreme. Manuf. 2025, 7, 062007.

[30] S. Qu , S. Gao , L. Wang , J. Ding , Y. Lu , Y. Wen , X. Qu , B. Zhang , X. Song , Addit. Manuf. 2024, 85, 104166.

[31] L. Liu , D. Wang , T. Wang , C. Han , Y. Li , H. Tan , W. Zhou , X. Yan , L. Lei , Y. Yang , Int. J. Mach. Tools Manuf. 2025, 205, 104236.

[32] Y. Wen , X. Wu , A. Huang , R. L. Narayan , P. Wang , L. Zhang , B. Zhang , U. Ramamurty , X. Qu , Acta Mater. 2024, 264, 119572.

[33] Y. Guo , H. Su , H. Gao , Z. Shen , P. Yang , Y. Liu , D. Zhao , Z. Zhang , M. Guo , X. Tan , Int. J. Plast. 2024, 179, 104050.

[34] J. Fu , J. Ding , S. Qu , L. Zhang , M. Y. Wang , M. Fu , X. Song , Mater. Des. 2022, 111018.

[35] J. X. Yang , X. H. Chen , Y. X. Sun , J. F. Zhang , C. Feng , Y. M. Wang , K. Wang , L. Bai , Mater. Des. 2022, 218, 110683.

[36] R. Miralbes , S. Higuera , D. Ranz , J. A. Gomez , Mech. Adv. Mat. Struct. 2021, 29, 5142.

[37] M. Zhang , Y. Yang , D. Wang , C. Song , J. Chen , Mater. Des. 2019, 165.

[38] L. Zhang , S. Feih , S. Daynes , S. Chang , M. Y. Wang , J. Wei , W. F. Lu , Addit. Manuf. 2018, 23, 505.

[39] A. Hanssen , O. S. Hopperstad , M. Langseth , H. Ilstad , Int. J. Mech. Sci. 2002, 44, 359.

[40] B. McDonnell , V. Errico , P. Posa , A. Angelastro , A. Furman , E. O'Hara , S. L. Campanelli , N. Harrison , Addit. Manuf. 2024, 89, 104301.

[41] J. Yang , X. Chen , Y. Sun , J. Zhang , C. Feng , Y. Wang , K. Wang , L. Bai , Mater. Des. 2022, 218, 110683.

[42] I. Maskery , N. Aboulkhair , A. Aremu , C. Tuck , I. Ashcroft , R. D. Wildman , R. Hague , Mater. Sci. Eng.: A 2016, 670, 264.

[43] L. Yang , R. Mertens , M. Ferrucci , C. Yan , Y. Shi , S. Yang , Mater. Des. 2019, 162, 394.

[44] Y. Wen , B. Zhang , R. L. Narayan , P. Wang , X. Song , H. Zhao , U. Ramamurty , X. Qu , Addit. Manuf. 2021, 20, 101926.

[45] J. Ding , Q. Zou , S. Qu , P. Bartolo , X. Song , C. C. Wang , CIRP Ann. Manuf. Technol. 2021, 70, 167.

[46] A. Zadpoor , J. Sinke , R. Benedictus , Tailor‐Welded Blanks Adv. Manufact. 2011, 68.

[47] C. Bonatti , D. Mohr , Int. J. Plast. 2017, 92, 122.

