# Supplementary material for: Material‐Gradient Enabled Enhancement of Strength and Strain‐Hardening of Lattice Structures
Source: Adv Sci (Weinh). 2025 Oct 7;12(48):e11185. doi: 10.1002/advs.202511185 (PMC12752590; doi:10.1002/advs.202511185)
Supplement: Supplementary file 1 — Supporting Information [file ADVS-12-e11185-s001.docx]

**Supporting information**

**Material-Gradient Enabled Enhancement of Strength and Strain-hardening of Lattice Structures**

Junhao Ding ^1, 2^, Yaojie Wen ^1^, Qianhua Wang ^1^, Shuo Qu ^2^, Xu Song ^2,^ *, Baicheng Zhang ^1, 3,^*

^1^ Beijing Advanced Innovation Center for Materials Genome Engineering, Institute for Advanced Materials and Technology, University of Science and Technology Beijing, Beijing 100083, P. R. China

^2^ Department of Mechanical and Automation Engineering, Chinese University of Hong Kong, Shatin, Hong Kong, 999077, P. R. China

^3^ Beijing laboratory of modern transportation metal materials and processing technology, University of Science and Technology Beijing, Beijing 100083, P. R. China

J.D., Y.W. contributed equally to this work.

^*^ Corresponding Author:

E–mail: xsong@mae.cuhk.edu.hk; zhangbc@ustb.edu.cn


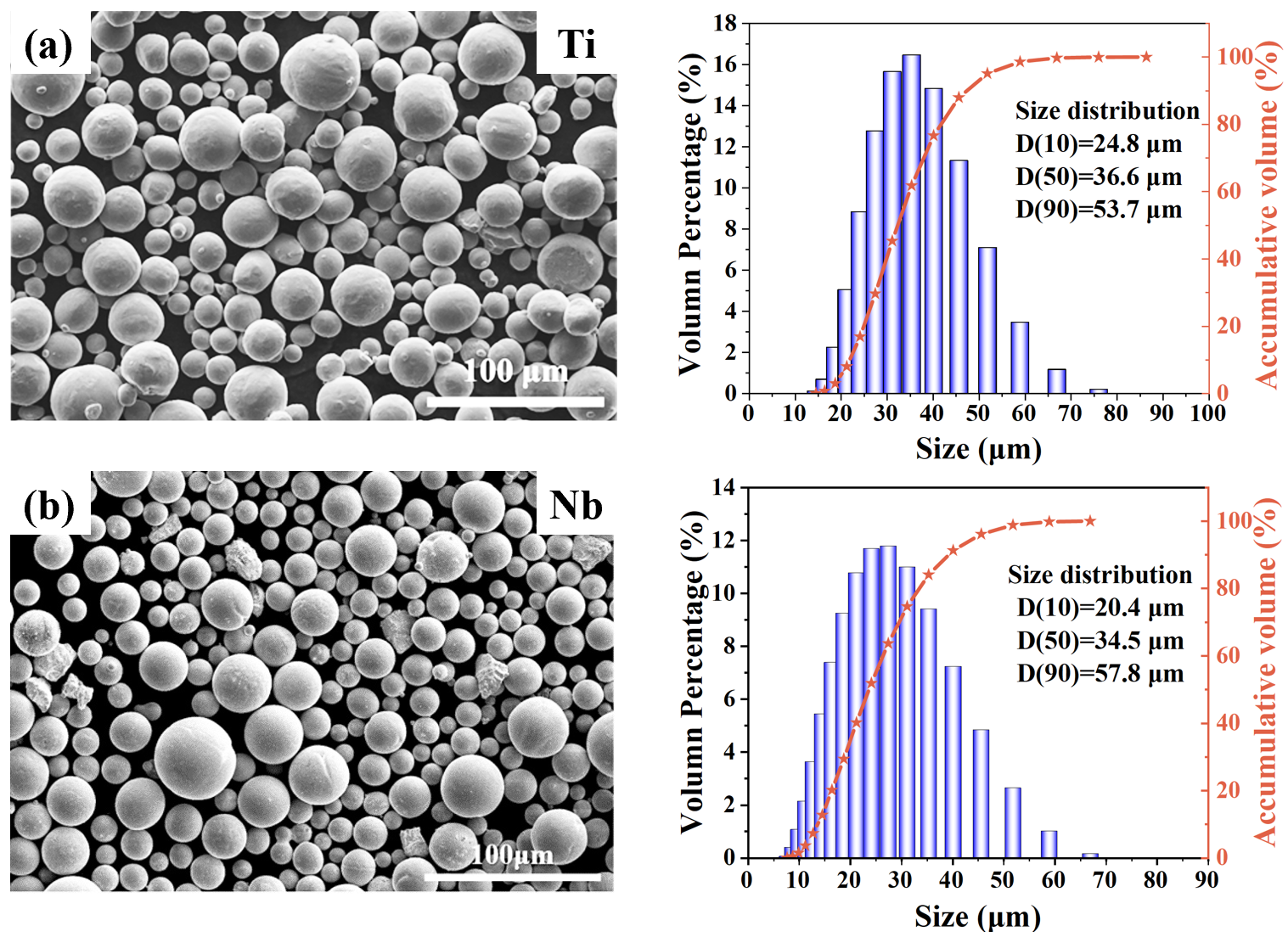


Fig. S1 Morphology of powder: (a) Ti alloy powder, (b) Nb alloy powder.


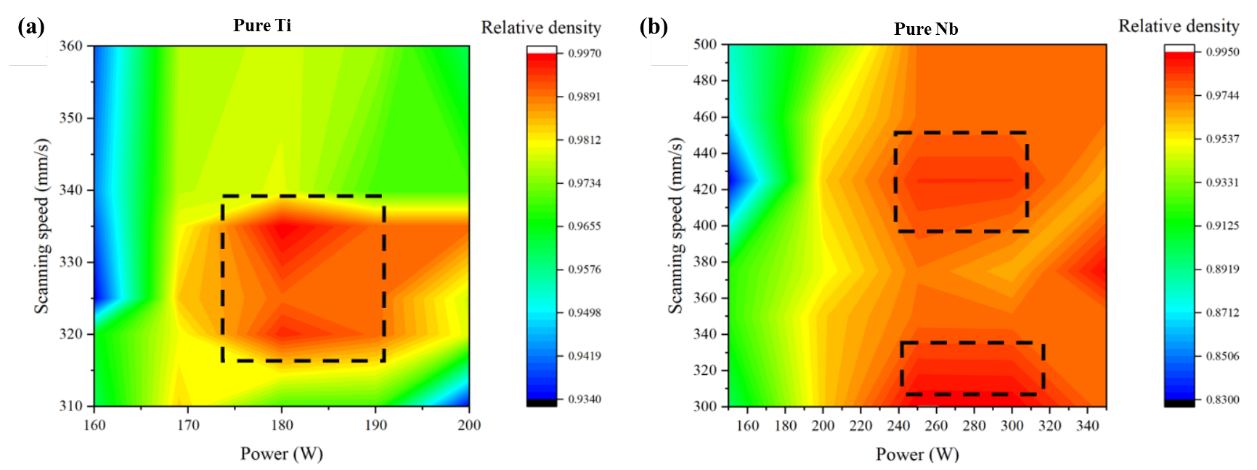


Fig. S2 Relative density of as-printed part using different printing parameters: (a) Ti; (b) Nb.





Fig. S3 Scanning speed and laser power at different Nb contents.


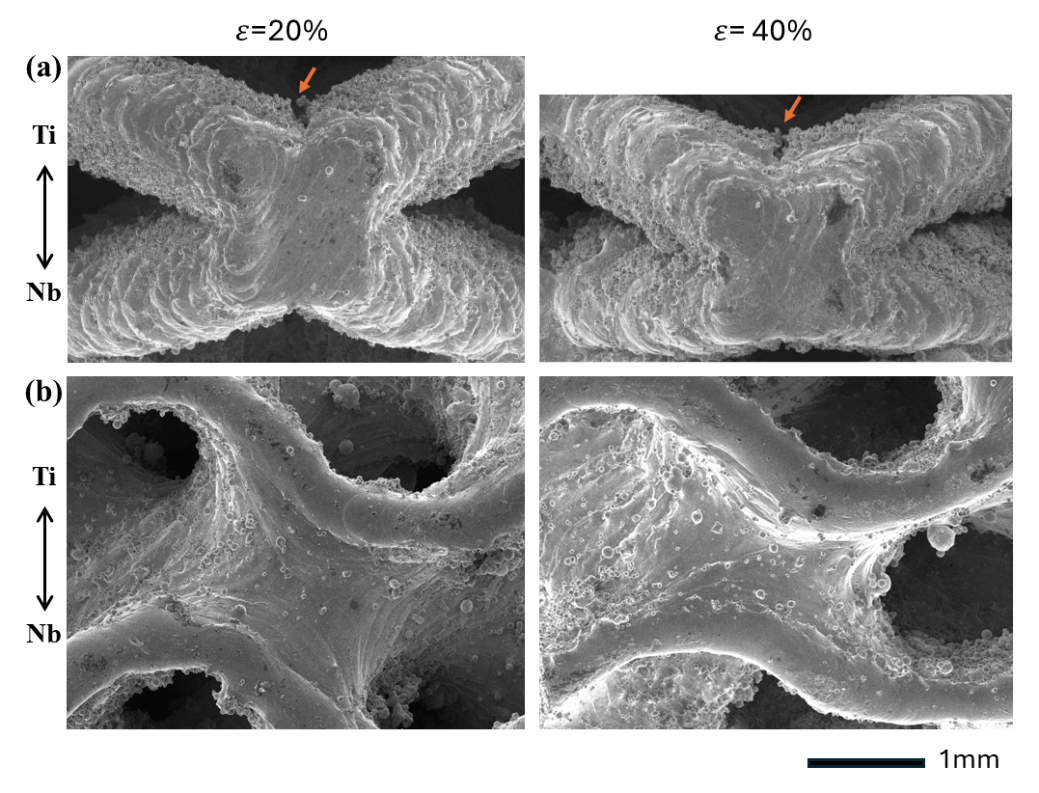


Fig. S4 Morphology of lattices at different compression strains of 20% and 40%: (a) BCC truss; (b) TPMS.


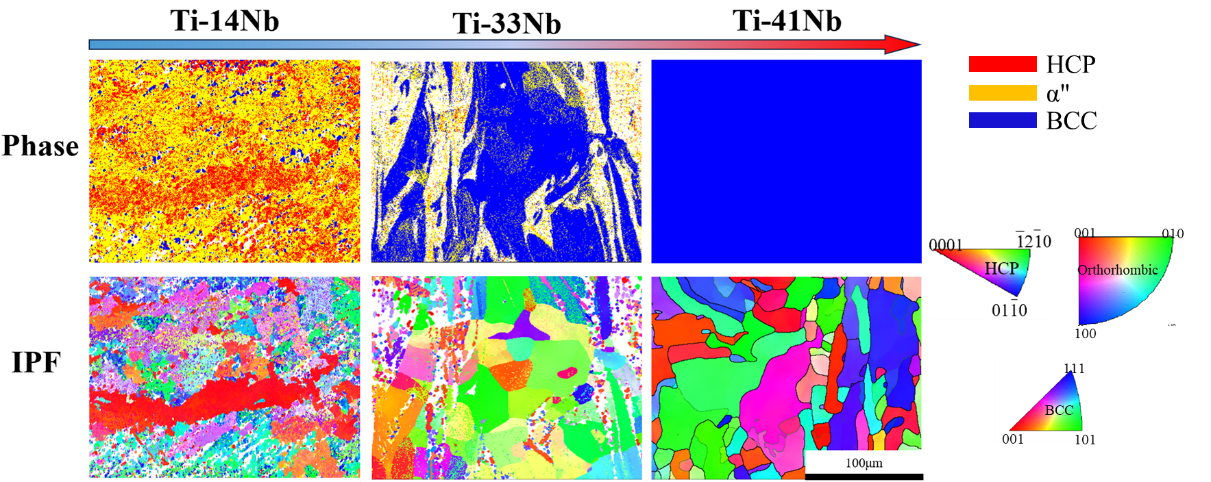


Fig. S5 The EBSD results of Ti-Nb alloy with different compositions.


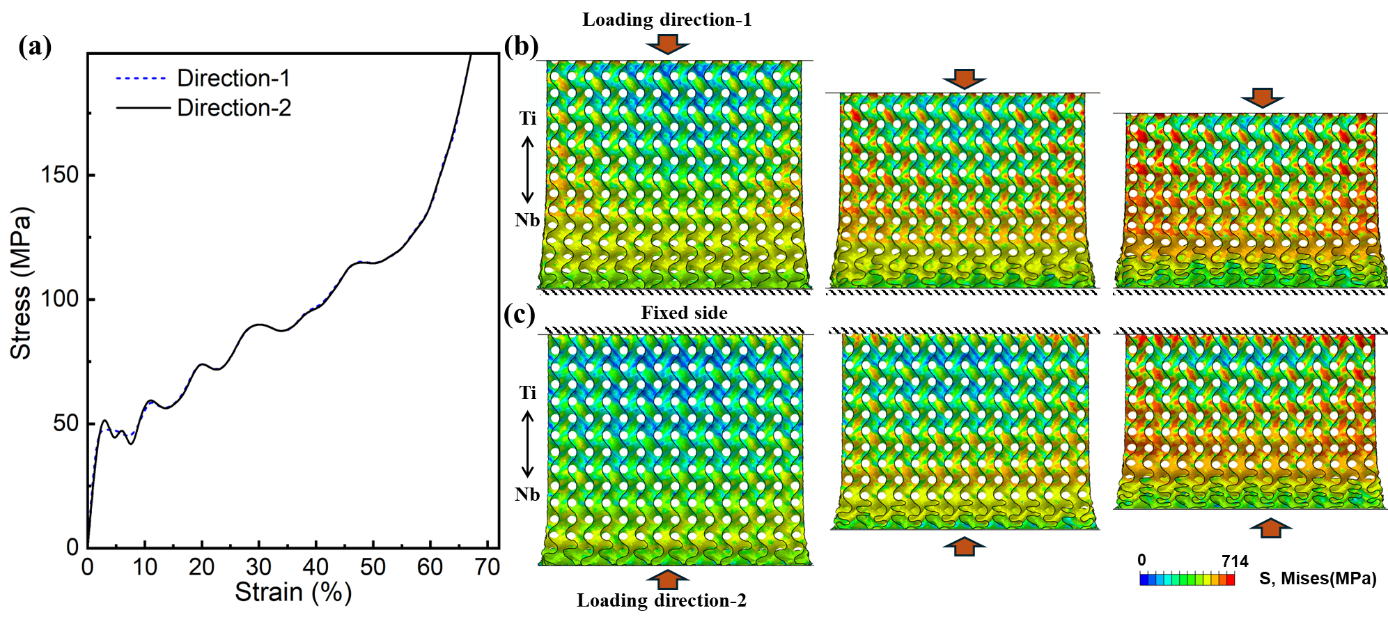


Fig. S6 Deformation behavior under different loading directions: (a)Stress-strain curves; (b, c) Collapse behavior.


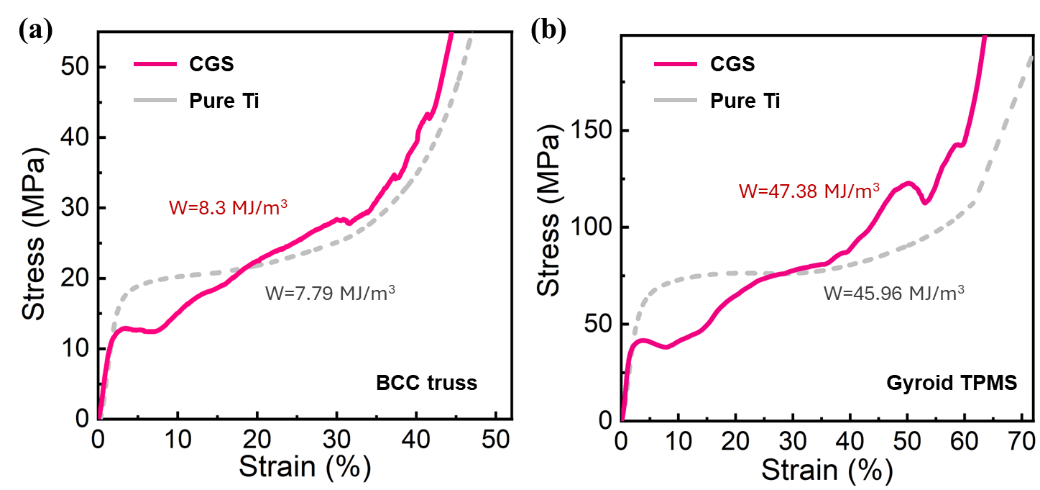


Fig. S7 The comparison between CGS and pure Ti lattices: (a) BCC truss, (b) Gyroid TPMS.


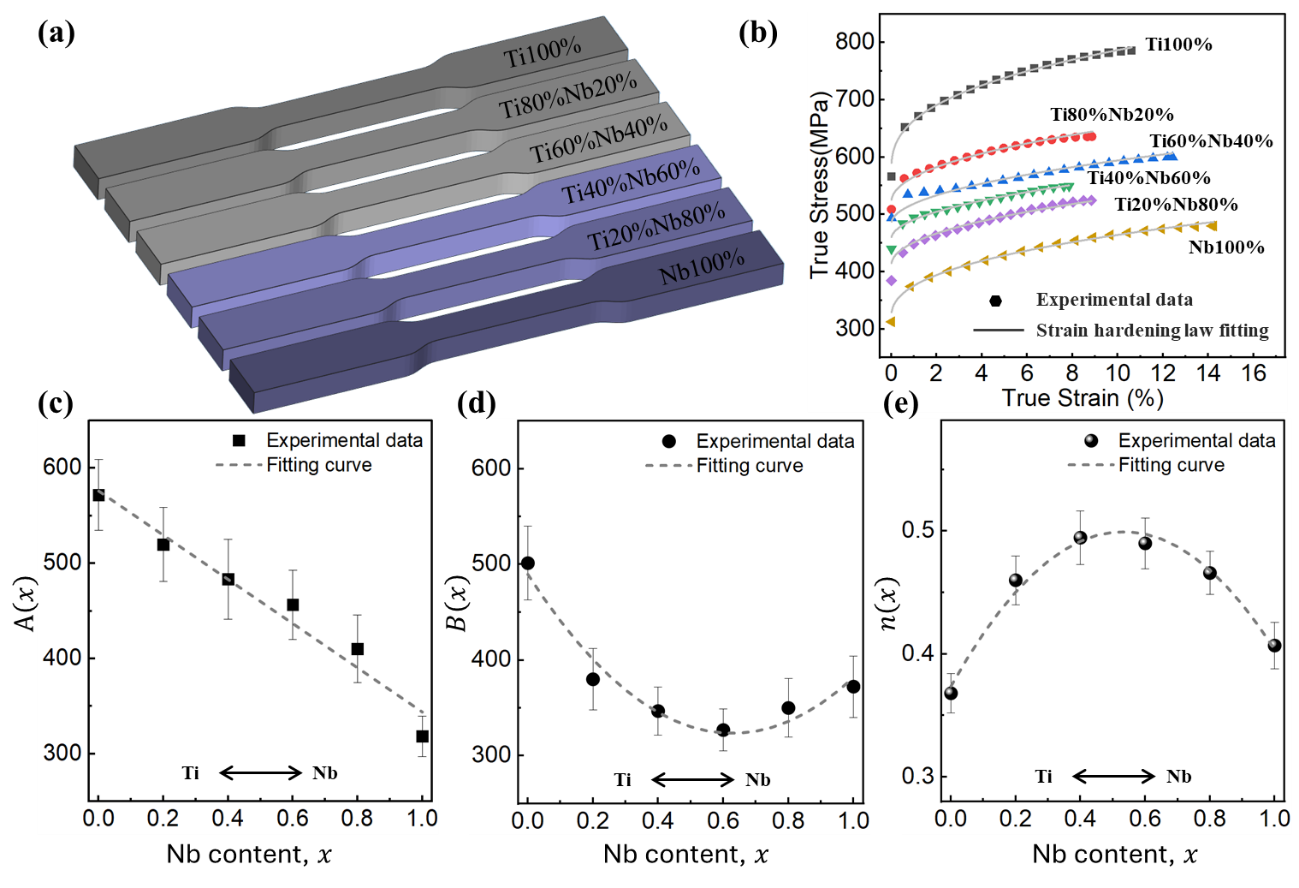


Fig. S8 Determination of strain hardening parameters: (a, b) Fitting parameters in Eq. (6), (c-e) Relationship between *A*, *B*, *n* and composition.


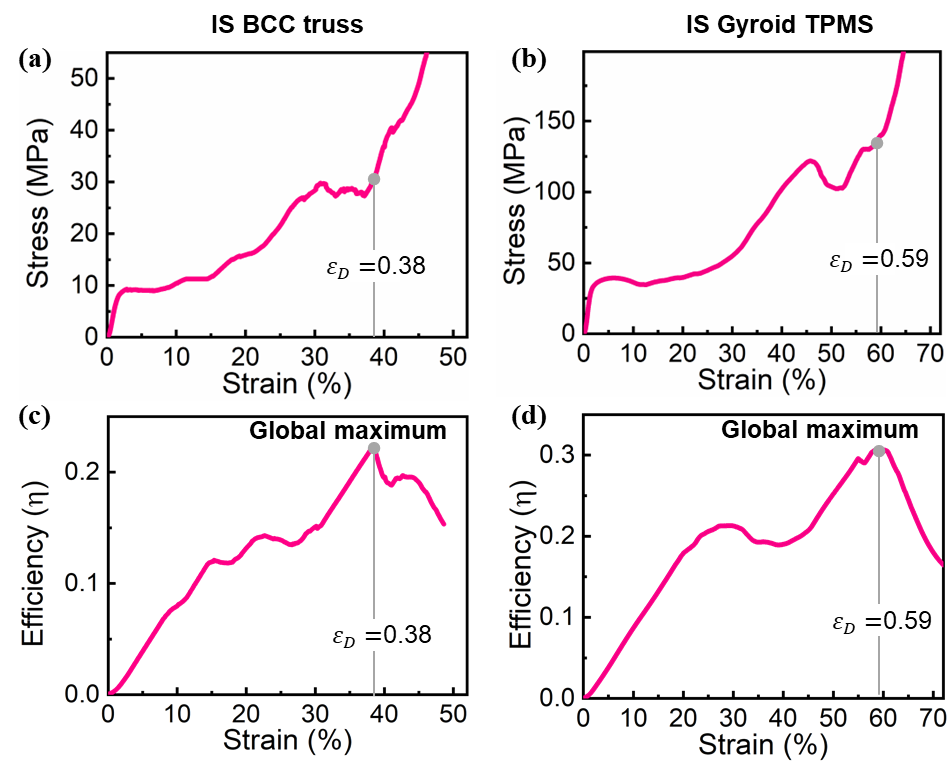


Fig. S9 Evaluation of mechanical energy absorption capability via compressive testing curves: (a, b) Engineering stress-strain curves; (c, d) Energy absorption efficiency curves.


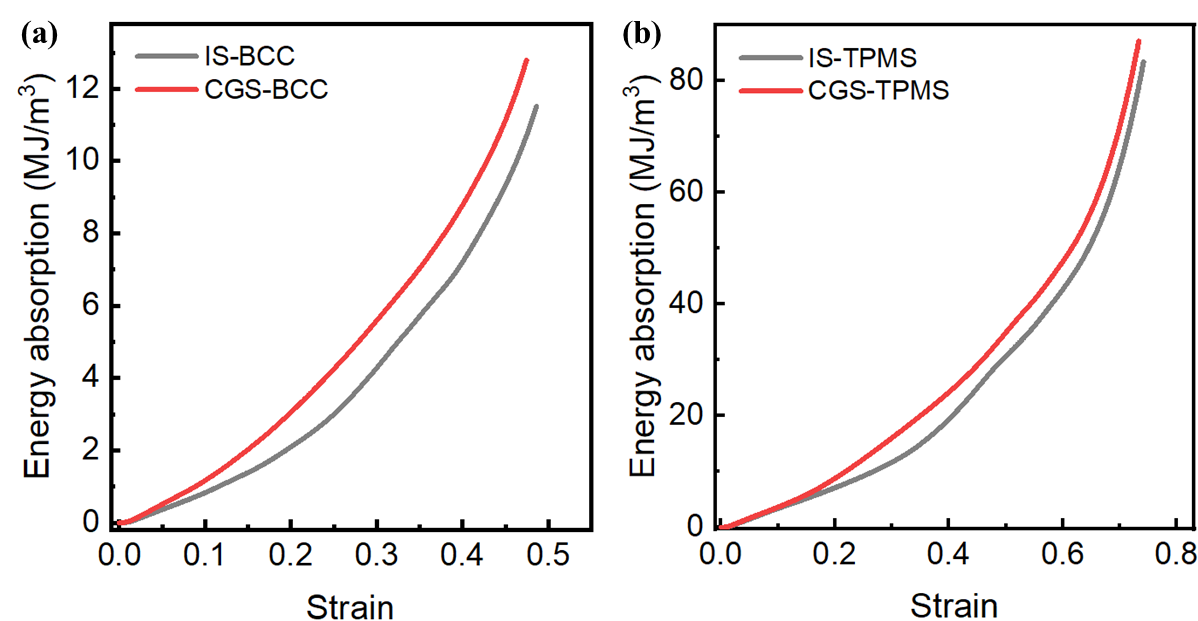


Fig. S10 Energy absorption of Ti-Nb BCC Truss lattice structures(a) BCC truss; (b) Gyroid TPMS.

**
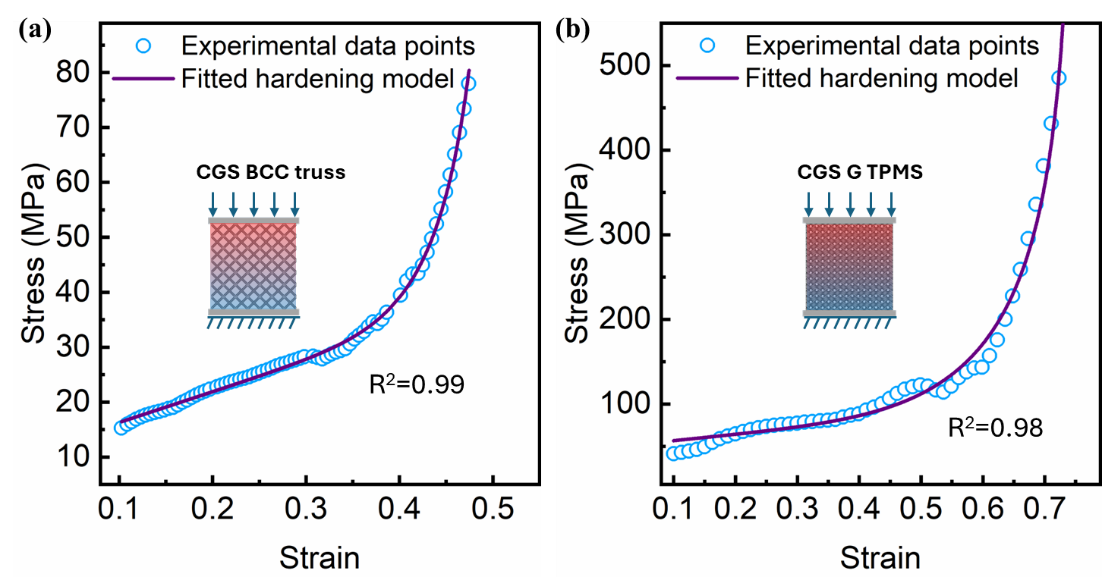
**

Fig. S11 Curve fits of the experimental data and strain-hardening analytic model: (a) CGS truss, (b) CGS TPMS.


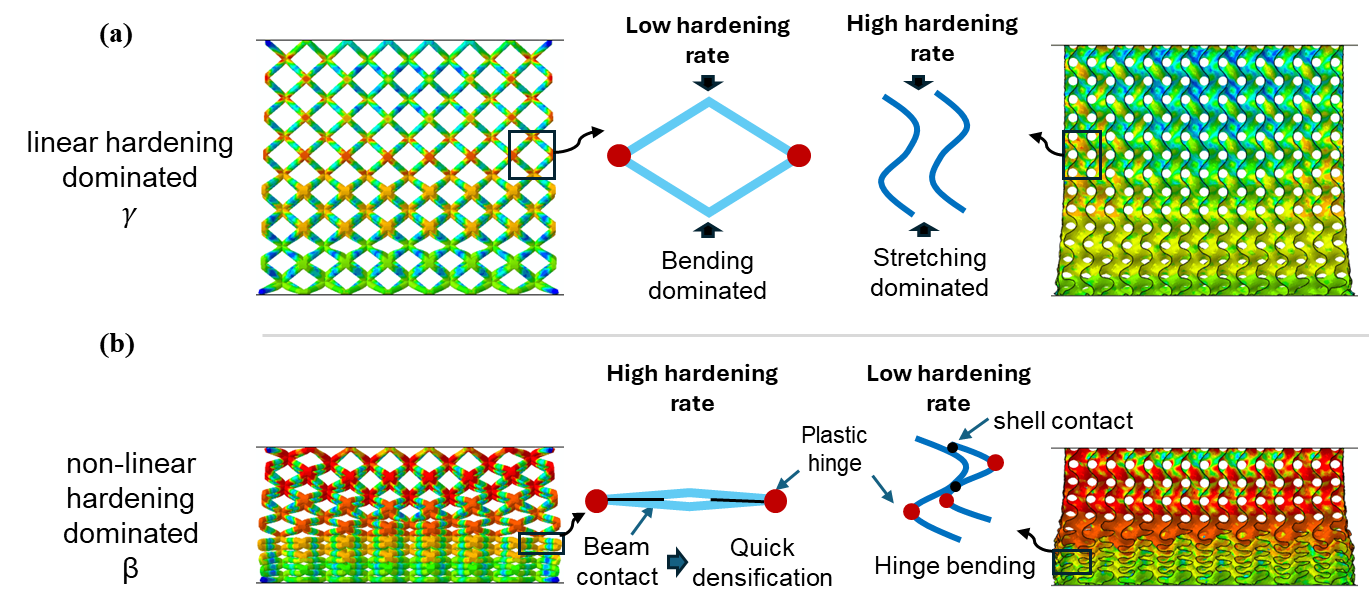


Fig. S12 Illustration of hardening behavior of truss and TPMS at different deformation stages.


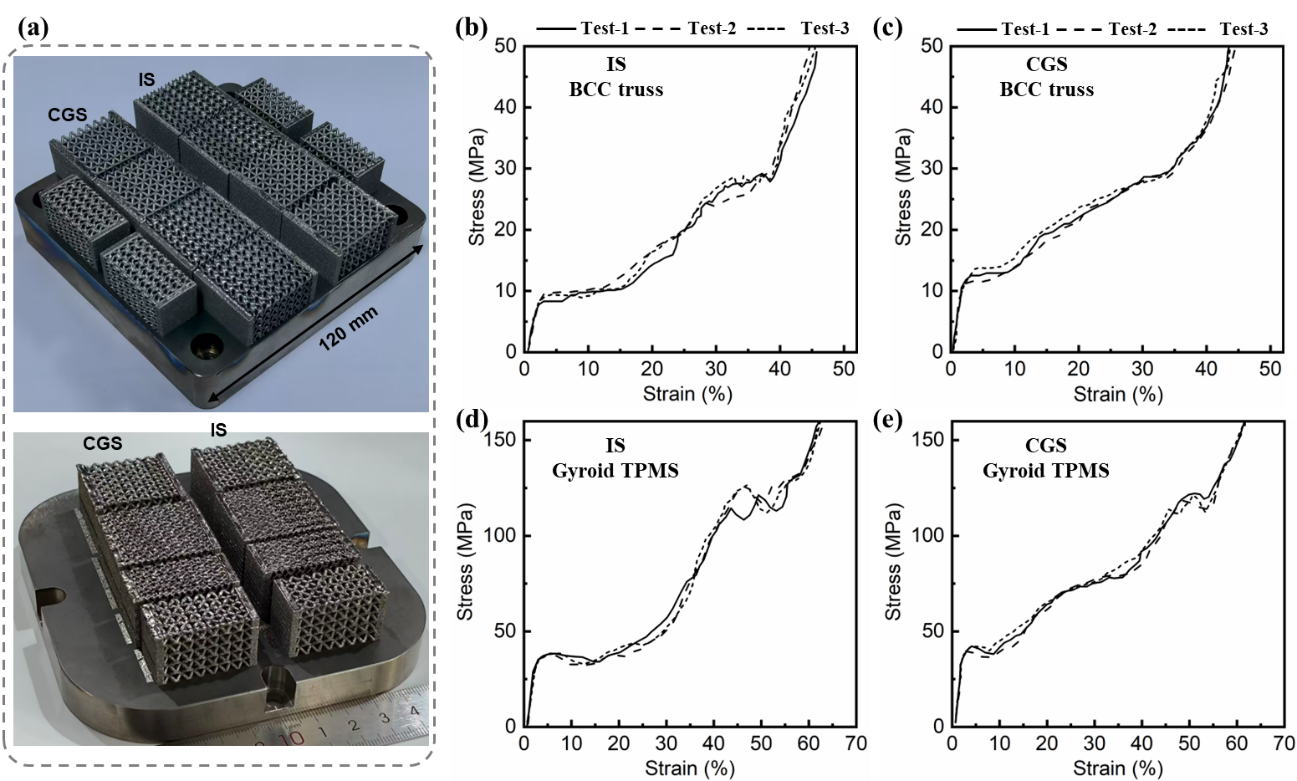


Fig. S13 Validation of reliability and reproducibility: (a) As-printed lattice structures; (b, c) stress–strain curves of IS and CGS BCC truss lattices; (d, e) stress–strain curves of IS and CGS Gyroid TPMS lattices.

Table S1 Dimensional size and density of as-printed lattices

| Lattice type | Gradient connection type | Measured mass (g) | Measured dimensions  *x* × *y* × *z* (mm^3^) | Relative density |
| --- | --- | --- | --- | --- |
| BCC  Truss | Ti-Nb IS | 23.4175 | 30.50×28.25×13.21 | 26.8% |
|  | Ti-Nb CGS | 24.2929 | 30.48×28.34×13.29 | 26.7% |
| Gyroid  TPMS | Ti-Nb IS | 27.4336 | 32.10×28.26×13.09 | 26.1% |
|  | Ti-Nb CGS | 27.4470 | 32.10×28.27×13.31 | 26.2% |

Table S2 Constants in strain hardening law.

| Material | A(MPa) | B(MPa) | *n* |
| --- | --- | --- | --- |
| Ti | 571.9±18.7 | 504.4±19.2 | 0.368±0.008 |
| Ti80%Nb20% | 519.7±19.4 | 380.2±16.1 | 0.460±0.010 |
| Ti60%Nb40% | 483.1±21.0 | 346.7±12.5 | 0.495±0.011 |
| Ti40%Nb60% | 456.6±18.1 | 326.9±10.9 | 0.490±0.010 |
| Ti80%Nb20% | 410.2±17.8 | 350.0±15.3 | 0.466±0.009 |
| Nb | 318.3±10.7 | 372.3±16.1 | 0.407±0.009 |

Table S3 The relationship between parameter *A*, *B*, *n* and element composition *x*.

| Parameter | *p_1_* | *p_2_* | *p_3_* |
| --- | --- | --- | --- |
| *A* | / | -231.9 | 579.9 |
| *B* | 421.4 | -529.3 | 489.7 |
| *n* | -0.442 | 0.471 | 0.374 |

*p(x)=p_1_x^2^+p_2_x+p_3_; p* represents the *A, B,* and *n.*

Table S4 Constants in strain hardening models

| Lattices | $\sigma_{p}$ | $\gamma$ | $\alpha$ | $\beta$ |
| --- | --- | --- | --- | --- |
| CGS truss | 10.69±0.32 | 28.57±0.89 | 65.13+2.67 | 10.03±0.28 |
| CGS TPMS | 47.18±1.32 | 55.63±1.44 | 197.67±6.72 | 5.04±0.165 |

Table S5 Summary of the other types of multi material 3D printing technologies

|  | In-situ powder mixing (LPBF) [27] | Selective powder removal for LPBF [29,31] | Interlayer material change for LPBF [28] | Directed energy deposition [25] | |
| --- | --- | --- | --- | --- | --- |
| Material Distribution Flexibility | Z-direction only with gradient [0.5] | Limited 3D, no smooth gradient [0.75] | Z-direction only, no gradient [0.25] | Full 3D control [1.0] | |
| Geometric design freedom | High (same as standard LPBF) [0.9] | High (same as standard LPBF) [0.9] | High (same as standard LPBF) [0.9] | Limited for complex geometries [0.75] | |
| Adaptive process control | Limited parameter control [0.75] | Limited parameter control [0.75] | Limited parameter control [0.75] | Flexible toolpath & laser control [1] | |
| Mechanical property tailoring | Limited by Z-direction design [0.75] | Interface defects may occur [0.75] | Z-direction only and interface issues [0.67] | Tailored via path & building direction [0.92] | |
| Resolution control | Spot size: 50-80 μm (standard LPBF) [0.75] | Spot size: 50-80 μm (standard LPBF) [0.75] | Spot size: 50-80 μm (standard LPBF) [0.75] | | Spot size: 250-2000 μm [0.25] |

*Values in brackets (e.g. [0.8]) represent a relative performance score from 0 to 1. The scoring criteria is as follows:*

**(1) Material Distribution Flexibility:** The ability to spatially control material placement and gradient transitions during the printing process.

Scoring Criteria:

[1.0] Full 3D control with smooth, continuous material gradients;

[0.75] Partial 3D control with sharp interfaces, or partial 2D control with smooth gradients;

[0.5] Partial 2D control or 1D (Z-direction only) control with smooth gradients;

[0.25] 1D (Z-direction only) control with sharp interfaces or no smooth transition capability.

Our method score: 1.0 — Our in-house developed LPBF system enables full 3D spatial control of material composition with smooth gradient transitions, achieving the highest flexibility in material distribution.

**(2) Geometric design freedom:** The capability to fabricate complex architectures (e.g., lattices, TPMS structures) without restrictions imposed by the printing method.

Scoring Criteria:

[1.0] Full geometric freedom, equivalent to standard LPBF systems;

[0.75] Minor constraints in fabricating complex geometries (e.g., difficulty printing fine lattice features);

[0.5] Moderate constraints, not suitable for complex structures like lattices;

[0.25] Severe limitations — restricted to simple geometries only.

Our method score: 0.9 — Our system exhibits no significant limitations in printing complex geometries such as truss and TPMS lattices, achieving near-maximal geometric design freedom.

**(3) Adaptive process control:** The degree to which process parameters (e.g., laser power, scan speed, scanning path) can be locally adjusted during the build process.

Scoring Criteria:

[1.0] Fully customizable control over all laser track parameters, including toolpath, power, and speed;

[0.75] Partial or software-limited control with flexible scanning strategies and some parameter adjustability;

[$\leq$0.5] Basic parameter adjustment only.

Our method score: 1.0 — Our custom-developed LPBF platform supports full adaptive control over laser toolpaths and processing parameters, enabling precise, location-specific optimization during printing.

**(4) Mechanical property tailoring:** Ability to fine-tune mechanical performance.

Scoring Criteria:

The performance of as-printed samples is typically influenced by a combination of material composition, process parameters, and geometric design. Therefore, this factor is approximated as the average score of Material Distribution Flexibility, Adaptive Process Control, and Geometric Design Freedom.

**(5) Resolution control:** Minimum achievable feature size in the printed structure, which is mainly determined by the laser spot size.

Scoring Criteria:

[1] Spot size is smaller than 50 μm;

[0.75] Spot size is within 50~100 μm;

[0.5] Spot size is larger than 100 μm;

[0.25] Spot size is larger than 100 μm.

Our method score:1.0 — Our LPBF system utilizes a high-resolution 30 μm laser spot, enabling precise fabrication of fine features such as thin walls and narrow struts in lattice structures.
